# KDM6A promotes diabetic retinopathy via H3K27me3-dependent ferroptosis in Müller cells

**DOI:** 10.1038/s41419-026-08816-9

**Published:** 2026-04-29

**Authors:** Yanjun Wen, Siyue Luo, Cheng Hu, Yikang Ji, Yulin Zhang, Xu Wang, Xin Huang, Yan Wei

**Affiliations:** 1 Department of Ophthalmology, Eye & ENT Hospital, Shanghai Medical College, Fudan University, Shanghai, China; 2 Shanghai Key Laboratory of Visual Impairment and Restoration (Fudan University), Shanghai, China; 3 NHC Key Laboratory of Myopia (Fudan University), Key Laboratory of Myopia, Shanghai Key Laboratory of Visual Impairment and Restoration (Fudan University), Chinese Academy of Medical Sciences, Shanghai, China; 4 Department of Oral and Maxillofacial-Head and Neck Oncology, Shanghai Ninth People’s Hospital, Shanghai Jiao Tong University School of Medicine, Shanghai, China; 5 College of Stomatology, Shanghai Jiao Tong University; National Center for Stomatology; National Clinical Research Center for Oral Disease, Shanghai, China; 6 Shanghai Key Laboratory of Stomatology & Shanghai Research Institute of Stomatology, Shanghai, China

**Keywords:** Diabetes complications, Necroptosis

## Abstract

Diabetic retinopathy (DR), a leading cause of blindness, is driven by pathological angiogenesis and vascular leakage, but the underlying epigenetic mechanisms remain poorly defined. Here, we identify the histone demethylase KDM6A as a critical epigenetic regulator promoting DR pathogenesis via Müller cells. KDM6A expression is significantly elevated in human DR retinas and diabetic mouse models. Intravitreal silencing of *Kdm6a* ameliorates retinal vascular dysfunction, reducing neovascularization, leakage, and acellular capillaries while restoring endothelial tight junctions. Single-cell RNA sequencing revealed that *Kdm6a* knockdown specifically suppresses VEGF signaling and attenuates ferroptosis. Müller cell-specific *Kdm6a* overexpression exacerbated DR vascular pathology, while in vitro co-cultures confirmed that KDM6A in Müller cells induces endothelial dysfunction. Mechanistically, KDM6A demethylates H3K27me3 at the promoters of pro-ferroptotic genes (e.g., *Tfr1*, *Cybb*, *Atg7*), thereby promoting ferroptosis in Müller cells under high glucose conditions. Crucially, pharmacological or genetic inhibition of KDM6A mitigated high glucose-induced ferroptosis. Our findings establish KDM6A-mediated epigenetic control of Müller cell ferroptosis as a fundamental regulator of diabetic retinal vasculopathy and nominate KDM6A as a promising therapeutic target for DR.

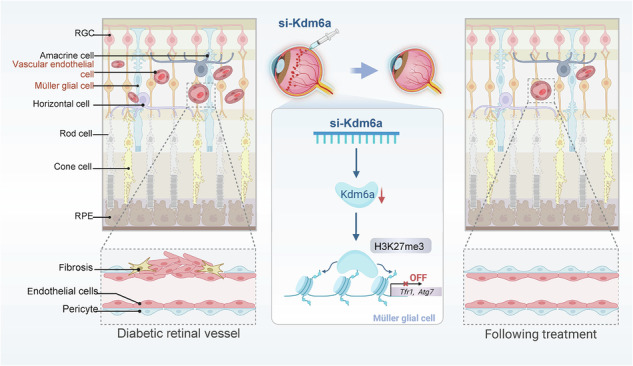

## Introduction

Diabetic retinopathy (DR) is the most common ocular complication of diabetes and the main cause of vision impairment and blindness in working age people [1]. For patients with diabetic macular edema, retinal laser photocoagulation, intravitreal injection of anti-vascular endothelial growth factor (VEGF) drugs, or corticosteroid drugs can be used. For patients with proliferative diabetic retinopathy (PDR), the most common treatment methods are retinal laser photocoagulation and vitrectomy [2]. Although existing treatment methods can maintain patients’ vision to a certain extent, they all have limitations in treatment [3]. For example, over 30% of patients with anti-VEGF therapy are ineffective, while a considerable portion of patients who initially respond will develop resistance or treatment resistance as the number of injections increases. Therefore, understanding the pathogenesis of DR, suppressing VEGF production at its source, and developing more effective targeted therapies remain key research priorities in DR.

Müller cells, the primary glial cells in the retina, play key roles in nutrition, neuronal protection, regulating retinal ion and water homeostasis, and forming the blood-retinal barrier [4]. Especially, it plays an important role in the occurrence and development of diabetic retinopathy [5]. In the early stages of disease development in DR animal models, Müller cells produce approximately 50% VEGF [6], indicating that activation of Müller cells is a necessary pathogenesis of DR. Notably, hyperglycemia-induced reactive oxygen species (ROS) in Müller cells upregulate VEGF [7]. Since ferroptosis is a form of cell death driven by lipid ROS and shares this oxidative stress axis, we hypothesize that it contributes to Müller cell dysfunction and pathological VEGF secretion in DR. While ferroptosis is documented in other retinal cells [8–10], its role in Müller cells and potential crosstalk with VEGF-driven angiogenesis remains unstudied. Whether it drives Müller cell activation and pathological VEGF release, thereby contributing to vascular dysfunction and angiogenesis, remains largely unexplored.

Lysine-specific demethylase 6A (KDM6A), a H3K27Me3-specific demethylase, activates the expression of numerous important target genes [11–13]. KDM6A regulates ferroptosis in a HIF-independent way [14]. Despite its gene-regulatory role, the development of anti-diabetic drugs targeting KDM6A remains largely unexplored. In the retinas of streptozotocin (STZ)-induced diabetic mice, Kdm6a expression is significantly upregulated at both the protein and mRNA levels. While KDM6A has been implicated in other retinal cell types in DR [15], its specific role in Müller cells, particularly concerning ferroptosis and VEGF-driven vasculopathy, remains unknown.

In this study, we aim to investigate how KDM6A promotes pathological angiogenesis in Müller cells during DR progression and to explore the therapeutic potential to interfering KDM6A mRNA in Müller cells. Our findings not only reveal a novel epigenetic-metabolic axis in DR but also provide a translatable therapeutic strategy to potentially inhibit angiogenesis by restoring iron homeostasis.

## Result

### Silencing kdm6a suppresses aberrant vascular dysfunction in diabetic retina

**Fig. 1:**
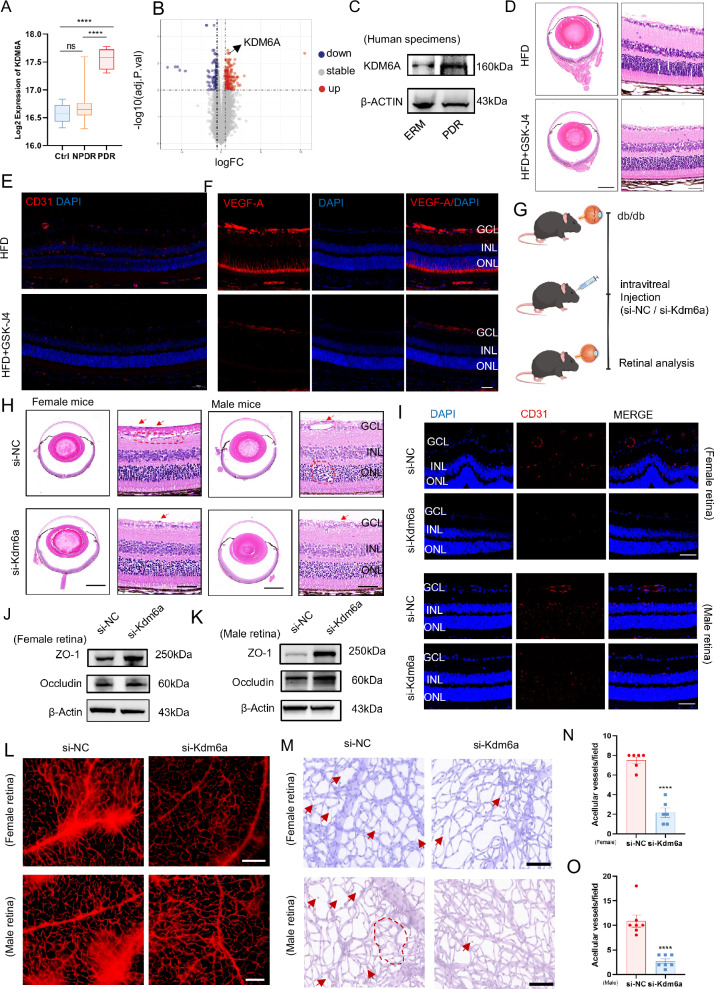
KDM6A downregulation alleviates diabetic retinopathy-associated vascular dysfunction. **A** Box plot of *KDM6A* mRNA expression (log_2_(TPM + 1)) from RNA-seq analysis of patient retinal tissues: Ctrl (*n* = 8), NPDR (*n* = 18), PDR (*n* = 5). **B** Volcano plot of differentially expressed genes (DEGs) in retinal tissues from PDR patients (*n* = 5) versus Ctrl subjects (*n* = 8) (logFC>0.5 or <0.5, adj.*p*.value < 0.05). **C** Western blot analysis of KDM6A protein levels in PVR from ERM or PDR patients. *n* = 3. **D** H&E staining of retinal sections from vehicle and GSK-J4-treated HFD mice (*n* = 3). Scale bar 50 μm. **E** Representative immunofluorescence images show retinal cross-sections from high-fat diet-fed (HFD) mice treated with either vehicle or GSK-J4 (*n* = 3), stained for CD31 (red) and DAPI (blue). Scale bar: 1 mm and 50 μm. **F** Representative immunofluorescence images of retinal cross-sections stained for VEGF-A (red) and DAPI (blue) in control and GSK-J4-treated (*n* = 3) mice. Scale bar: 50 μm. **G** Schematic diagram of the animal model experimental design. Created with BioGDP.com. **H** H&E staining of retinal sections from si-NC- and si-Kdm6a-treated db/db mice (both sexes, *n* = 3). Red arrow indicates abnormal nuclear morphology in the inner retinal layer. Red circle highlights pathological vascular dilation and disorganized ONL structure. Scale bar: 1 mm and 50 μm. **I** Retinal vascular analysis by CD31 immunofluorescence in db/db mice following intravitreal siRNA administration (both sexes, *n* = 3). Scale bar 50 μm. **J**, **K** Western blot analysis of ZO-1 and Occludin protein levels in the retinas of db/db mice receiving si-Kdm6a or si-NC intravitreal administration (both sexes, *n* = 3). **L** Vascular leakage was visualized using Evans blue in whole-mounted retinas. (both sexes, *n* = 3). Scale bar 20 μm. **M** PAS staining of retinal trypsin digestion visualizing retinal vasculature and acellular capillaries (red arrowheads) in db/db mice (both sexes) following intravitreal siRNA administration (*n* = 3). Scale bar 100 μm. **N**, **O** Quantification of acellular capillaries demonstrating decreased vascular pathology in KDM6A-deficient retinas (both sexes, *n* = 3). Data presented as mean ± SEM; ****p < 0.0001, one-way ANOVA followed by Tukey’s post hoc test and unpaired two-tailed Student’s t-test. GCL ganglion cell layer, INL inner nuclear layer, ONL outer nuclear layer.

Diabetic conditions trigger epigenetic alterations in the retina, leading to dysregulated expression of key genes, such as those encoding antioxidant enzymes, thereby contributing to the metabolic abnormalities and pathogenesis of diabetic retinopathy [16]. Given the role of histone modifications in this process, the histone demethylase KDM6A emerges as a potential regulator in diabetic retinopathy, likely influencing disease progression by modulating the expression of genes involved in oxidative stress response and metabolic pathways. To investigate whether KDM6A expression is elevated in diabetic retinopathy, we analyzed publicly available RNA-seq data from the Gene Expression Omnibus (GEO) database (GSE160306) [17], which includes human retina samples from normal controls, non-proliferative diabetic retinopathy (NPDR), and PDR patients. Differential expression analysis revealed that KDM6A levels were significantly higher in PDR retinal samples compared to both normal controls and NPDR groups. (Fig. 1A, B, and Supplementary Fig. 1A). Immunoblotting assay of proliferative membranes from PDR and epiretinal membranes (ERM) control samples showed that KDM6A protein expression was significantly elevated in PDR patients compared to the ERM group (Fig. 1C and Supplementary Fig. 1B). These data support KDM6A as a candidate contributing to the pathogenesis of DR.

To investigate the functional role of KDM6A in DR progression, we administered the KDM6A inhibitor GSK-J4 via intraperitoneal injection in a diet-induced mouse model of DR. Compared with vehicle-treated controls, GSK-J4 treatment significantly attenuated retinal pathological changes, as evidenced by reduced nuclear layer loss and improved structural integrity on hematoxylin and eosin (H&E) staining (Fig. 1D). CD31 is a commonly used biomarker for labeling vascular endothelial cells, and its upregulation reflects compensatory vascular hyperplasia caused by retinal ischemia [18, 19]. GSK-J4 treatment markedly reduced CD31-positive deep retinal capillaries (Fig. 1E and Supplementary Fig. 1C) and suppressed VEGF-A expression (Fig. 1F and Supplementary Fig. 1D), as evidenced by immunohistochemical staining. These findings suggest that pharmacological inhibition of KDM6A ameliorates the progression of diabetes-induced retinopathy.

To examine whether downregulation of Kdm6a ameliorates vascular abnormalities in a murine DR model, we intravitreally injected si-Kdm6a into right eye and si-NC into left eye of diabetic db/db mice. After 14 days, both eyes were collected and examined with morphological examination (Fig. 1G). Compared with si-NC-treated retina, si-Kdm6a ameliorated retinal vascular abnormalities in both male and female *db/db* mice. Furthermore, si-Kdm6a administration attenuated the loss of outer nuclear layer (ONL) thickness and improved retinal structural disorganization (Fig. 1H). Compared to intravitreal injection of si-NC-treated retina, si-KDM6A reduced neovascularization (Fig. 1I and Supplementary Fig. 1E), suggesting improved vascular stability. Evans Blue Assay is the gold standard method for quantitative blood-brain barrier disruption in DR research [20]. We found significantly reduced retinal vascular leakage of Evans blue in si-Kdm6a-treated db/db mice compared to si-NC treatment (Fig. 1L and Supplementary Fig. 1H). Retinal vascular characterization assays are a key technique for evaluating microvascular structural damage in DR research, to comprehensively reveal the vascular lesions of DR [21]. We found that the number of acellular capillary segments of si-Kdm6a-treated retina are significantly less than those of si-NC group in retinal vasculature digestion assays (Fig. 1M–O). Consistent with reduced vascular dysfunction, si-KDM6A elevated tight junction proteins (Fig. 1J, K and Supplementary Fig. 1F, G), indicating restored barrier integrity. Collectively, KDM6A knockdown preserves retinal vasculature by suppressing pathological angiogenesis and enhancing endothelial barrier function.

### Single-cell analysis reveals silencing Kdm6a in Müller cells reduces retinal angiogenesis

**Fig. 2:**
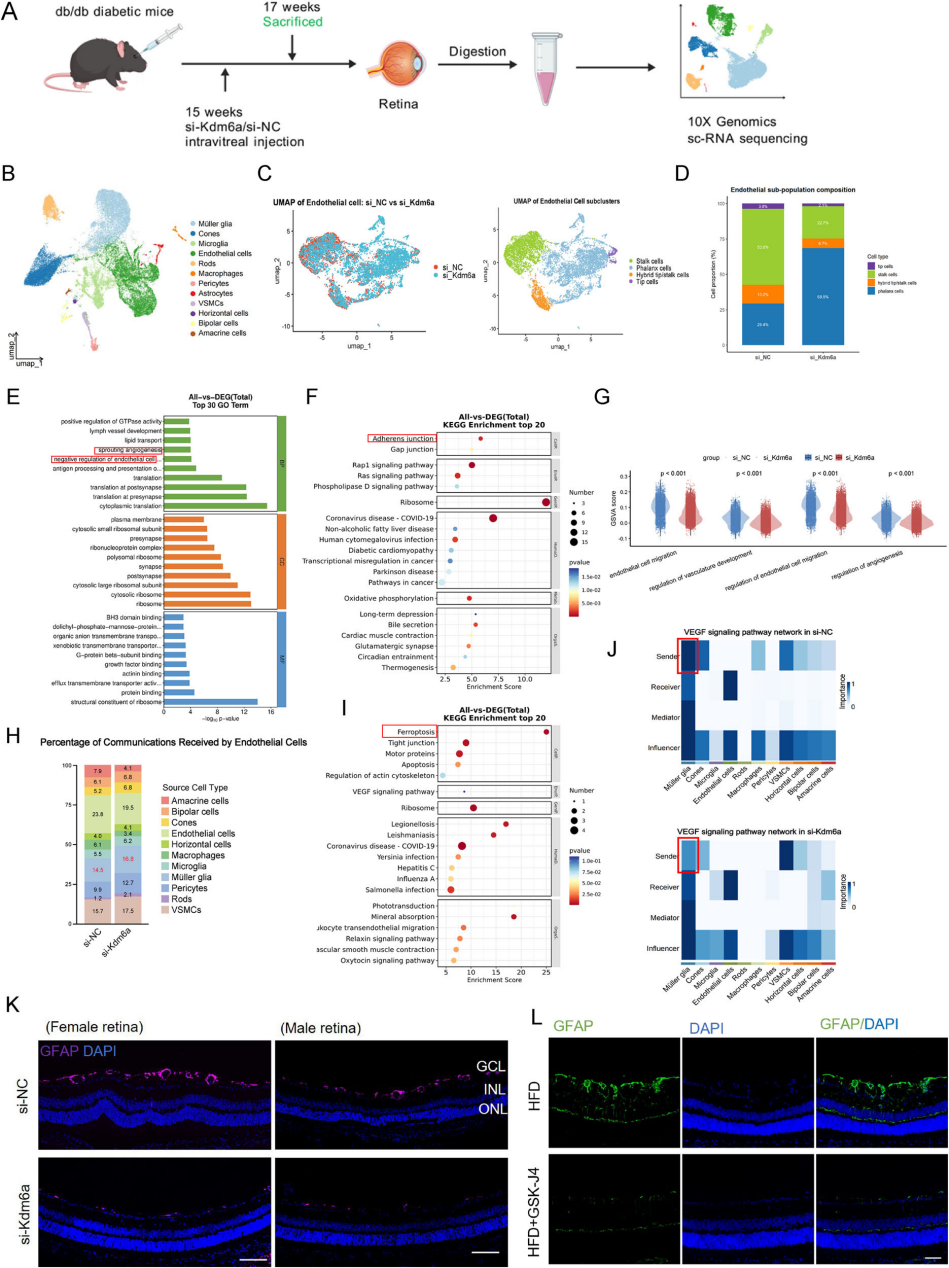
*Kdm6a* silencing induces endothelial remodeling and attenuated Müller cell VEGF Signaling in diabetic retinas. **A** Schematic of the experimental workflow: Intravitreal injections of si-Kdm6a or si-NC were administered to 15-week-old db/db mice. Retinas were harvested 2 weeks post-injection for single-cell RNA sequencing analysis. Created with BioGDP.com. **B** UMAP visualization of single-cell transcriptomes from scRNA-seq analysis in db/db mice following intravitreal injection with si-Kdm6a or si-NC. **C** UMAP visualization of endothelial cell clusters and subtypes in si-NC vs si-Kdm6a groups. Distinct endothelial subpopulations (tip, stalk, phalanx) are color-coded. **D** Bar plot showing endothelial subtype composition in si-NC vs si-Kdm6a groups from scRNA-seq data. Tip, stalk, and phalanx cell proportions are quantified as percentages of total endothelial cells. **E** Bar plot of Gene Ontology (GO) enrichment analysis performed on differentially expressed genes in endothelial cells. **F** Dot plot displaying the top 20 KEGG pathway enrichment results of differentially expressed genes in endothelial cells from scRNA-seq analysis. **G** Gene Set Variation Analysis (GSVA) scoring of endothelial migration and angiogenesis-related biological processes (BP) in si-NC vs si-Kdm6a groups. Key pathways—endothelial cell migration, regulation of vasculature development, regulation of endothelial cell migration, and regulation of angiogenesis—show significantly suppressed activity in si-Kdm6a. **H** Percentage bar plot of endothelial cell-received signaling from distinct cellular sources in si-NC vs si-Kdm6a groups, analyzed by CellChat (R package). **I** The top 20 dot plot of KEGG pathway enrichment analysis for differentially expressed genes (DEGs) in Müller cells between si-NC and si-Kdm6a groups. **J** Comparative analysis of VEGF signaling pathway activity in retinal cell types between si-NC and si-Kdm6a groups using CellChat. **K** GFAP immunofluorescence intensity in db/db mice following intravitreal siRNA administration. Scar bar = 50 μm, *n* = 3. **L** GFAP immunofluorescence intensity in HFD mice following GSK-J4 or vehicle treated. Scar bar = 50 μm, *n* = 3. Data = mean ± SEM, unpaired two-tailed Student’s t-test.

To further elucidate how KDM6A knockdown suppresses pathological retinal angiogenesis in diabetic retinopathy, we collected retinal samples 2 weeks post-injection and performed single-cell RNA sequencing (scRNA-seq) analysis (Fig. 2A). Following quality control and batch correction (Supplementary Fig. 2A), we obtained sequencing data from 12,030 cells in si-NC retinas, 16,940 cells in si-Kdm6a retinas. Using graph-based clustering, we grouped these cells based on their gene expression profiles. Subsequently, we annotated the resulting clusters via Uniform Manifold Approximation and Projection (UMAP) visualization based on established marker genes for specific retinal cell types [22]. We identified 12 distinct cell populations, including cones, rods, endothelial cells, astrocytes, bipolar cells, pericytes, microglia, Müller glia, horizontal cells, amacrine cells, vascular smooth muscle cells (VSMCs), macrophages (Fig. 2B). We further analyzed the mRNA expression of Kdm6a across all retinal cell types in our scRNA-seq data and found that Kdm6a expression was highest in three specific cell populations, including endothelial cells, Müller glia, and cones (Supplementary Fig. 2B). We observed a significant reduction in Kdm6a levels following intravitreal siKdm6a injection, confirming the efficacy of the treatment (Supplementary Fig. 2C).

In pathological angiogenesis, tip cells initiate aberrant sprouting through dysregulated matrix invasion, while stalk cells sustain abnormal vessel extension via excessive proliferation—both being essential drivers of neovascular disorders. Phalanx cells are quiescent endothelial cells that maintain vascular integrity through stable cell-cell junctions, exhibiting minimal migration, absent filopodia formation, and negligible proliferative activity [23]. Following scRNA-Seq analysis on endothelial subtyping, we observed predominant phalanx cell composition in si-Kdm6a-treated endothelia versus predominant tip/stalk cell populations in si-NC controls. (Fig. 2C, D and Supplementary Fig. 2D, E). Our Gene Ontology (GO) analysis demonstrated significant enrichment of terms associated with sprouting angiogenesis and negative regulation of endothelial cell processes (Fig. 2E). We specifically targeted angiogenesis-related biological processes for Gene Set Variation Analysis (GSVA), systematically quantifying endothelial cell migration, regulation of vasculature development, regulation of endothelial cell migration, and regulation of angiogenesis. KEGG pathway analysis further revealed significant enrichment of the adherens junction pathway (Fig. 2F). Comparative analysis demonstrated statistically significant attenuation of these terms in the *si-Kdm6a* group relative to *si-NC* (Fig. 2G). These results demonstrated that Kdm6a downregulation enhances endothelial stabilization through phenotypic switching.

To investigate cellular interactions with endothelial cells, we subsequently performed cell-cell communication analysis using the CellChat R package. CellChat analysis revealed an elevation in Müller cell-endothelial cell interactions following *si-Kdm6a* intravitreal injection compared to si-NC (16.8% vs. 14.5%) (Fig. 2H and Supplementary Fig. 2F), indicating that Müller cells with Kdm6a knockdown may suppress endothelial cell neovascularization in the diabetic retina. Müller cells are essential for retinal homeostasis as they are the radial glia spanning the entire retinal thickness [24, 25]. Therefore, we examined the RNA expression levels of *Kdm6a* in Müller cells within our scRNA-seq dataset. Compared with the si-NC group, the mRNA levels of *Kdm6a* were significantly decreased in the si-Kdm6a group (Supplementary Fig. 2G). KEGG pathway enrichment demonstrated that Kdm6a knockdown in Müller cells downregulated key pathways, including ferroptosis (Fig. 2I). Immunofluorescence analysis revealed that GFAP expression, a marker of reactive gliosis [26], was significantly suppressed in si-Kdm6a-treated db/db mice compared to si-NC group (Fig. 2K, Supplementary Fig. 2I). Similarly, GFAP expression of GSK-J4-treated mice was significantly lower than vehicle group (Fig. 2L, Supplementary Fig. 2J), supporting the alleviation of gliosis in diabetic retinopathy.

Given that Müller cell-derived VEGF accumulation under hyperglycemic conditions [6], we further examined the *VEGF* levels in Müller cells. ScRNA-seq analysis revealed that Müller cells in *si-Kdm6a*-treated db/db mouse retinas exhibited attenuated signaling sender activity and reduced VEGF mRNA levels compared to the si-NC group, suggesting their impaired capacity to mediate VEGF signaling in diabetic retinas (Fig. 2J and Supplementary Fig. 2H). These findings suggest that the observed improvement in retinal vascular abnormalities in the si-Kdm6a group may be attributed to the suppression of the VEGF pathway in Müller cells following Kdm6a downregulation.

### Müller cell-specific overexpression of Kdm6a exacerbates diabetic-induced retinal vascular dysfunction

**Fig. 3:**
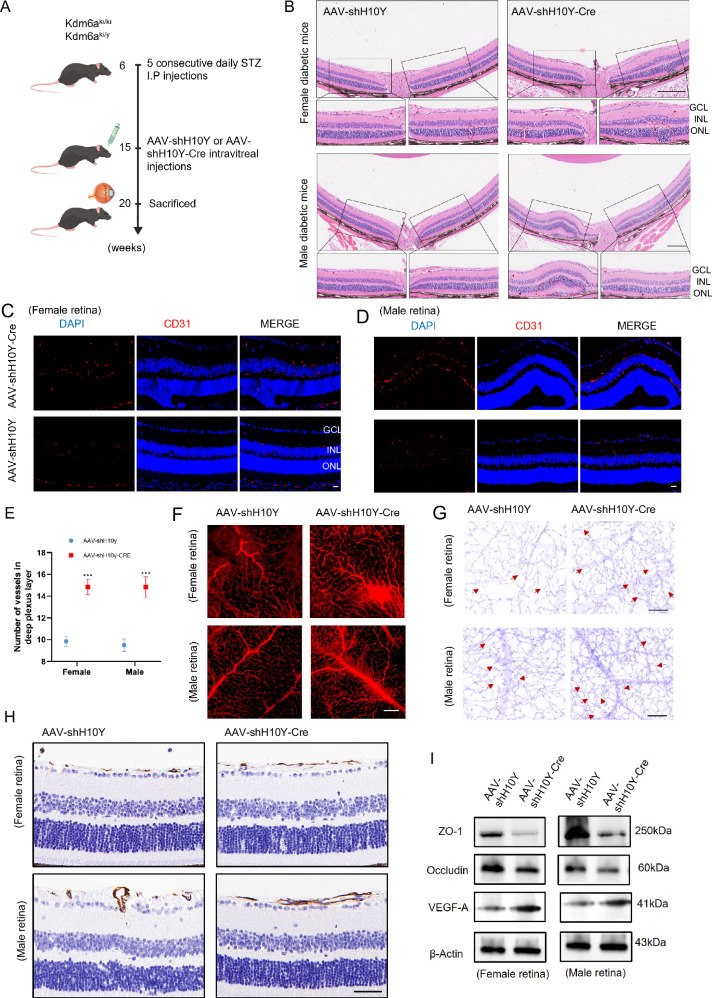
Kdm6a overexpression aggravates diabetic retinopathy by impairing vascular integrity in diabetic mouse models. **A** Schematic of KDM6A genetic modification in diabetic mouse models. Created with BioGDP.com. **B** H&E staining of retina sections in AAV-treated diabetic mice (both sexes, *n* = 3). Red circles denote structural abnormalities in the diabetic retinal tissue. The red arrow highlights luminal alterations in inner retinal blood vessels (narrowing or leakage), while the black arrow indicates disorganized cellular arrangement and reduced density in the ONL. Scale bar: 100 μm and 50 μm. **C**–**E** CD31 immunofluorescence analysis of retinal vasculature in AAV-treated diabetic mice (male and female, *n* = 3). Scale bar 50 μm. **F** Retinal vascular leakage was visualized using Evans blue in whole-mounted retinas of AAV-treated diabetic mice (both sexes, *n* = 3). Scale bar 20 μm. **G** Retinal vascular histology by PAS staining showing acellular capillaries (red arrowheads) in AAV-treated diabetic mice of both sexes (*n* = 3). Scale bar 100 μm. **H** Immunohistochemical staining with GFAP was performed on retinal sections from AAV-shH10y and AAV-shH10y-Cre diabetic mice (male and female, *n* = 3). Scale bar 50 μm. **I** Western blot analysis of ZO-1, Occludin, and VEGF-A protein levels in the retinas of AAV-shH10y and AAV-shH10y-Cre diabetic mice (male and female, *n* = 3). Data are presented as means ± SEM, unpaired two-tailed t-test. ***p < 0.001.

To establish whether the overexpression of Kdm6a in Müller cells is causal for DR, we generated male Kdm6a^ki/y^ and female Kdm6a^ki/ki^ transgenic mice and performed intravitreal injections of Müller cell-targeting AAV-shH10y-cre to Müller cell-specific *Kdm6a* knock-in (Fig. 3A). AAV-shH10y lacking Cre served as control. Based on the HE staining results, it can be observed that in both male and female mice from the AAV-shH10Y-cre group, the luminal structures of retinal inner layer vessels exhibited significant dilation (red arrows), accompanied by a more disorganized overall retinal architecture (red circles). Furthermore, the density of cellular arrangement in both the inner nuclear layer (INL) and outer nuclear layer (ONL) was notably reduced compared to AAV-shH10Y groups (black arrows) (Fig. 3B).

Next, we evaluated whether the overexpression of Kdm6a in Müller cells promotes angiogenesis in DR. Immunofluorescence analysis revealed a significant increase in vascular density within the deep plexus of retinal in the AAV-shH10Y-Cre group compared to AAV-shH10Y group (Fig. 3C–E). Furthermore, assessment of retinal vascular barrier integrity demonstrated significantly increased Evans blue extravasation in the AAV-shH10Y group, and this vascular leakage phenotype was further exacerbated following AAV-shH10Y-Cre intravitreal injections (Fig. 3F, Supplementary Fig. 3B). Simultaneously with Kdm6a overexpression in Müller cells, immunohistochemical analysis of GFAP demonstrated its localized high expression at the inner limiting membrane (Fig. 3H), indicating Müller cell gliosis. Additionally, we observed a significant increase in VEGF-A protein levels in the retinas of AAV-shH10Y-Cre group (Fig. 3I and Supplementary Fig. 3A). These data suggest that overexpression of *Kdm6a* in Müller cells disrupts retinal neuronal architecture, reduces nuclear layer density, and induces gliotic activation, implicating Müller cell-derived *Kdm6a* in driving neurodegenerative changes in diabetic retinopathy. Moreover, PAS staining results indicated a significant increase in the number of acellular capillaries in the AAV-shH10Y-Cre group, accompanied by a marked downregulation of tight junction proteins ZO-1 and Occludin expression compared to AAV-shH10Y group (Fig. 3G, I and Supplementary Fig. 3A, C). These findings collectively demonstrate that Kdm6a overexpression in retinal Müller cells exacerbates diabetes-induced retinal vascular abnormalities.

### Kdm6a increases VEGF production in Müller cells and promotes endothelial cell proliferation

**Fig. 4:**
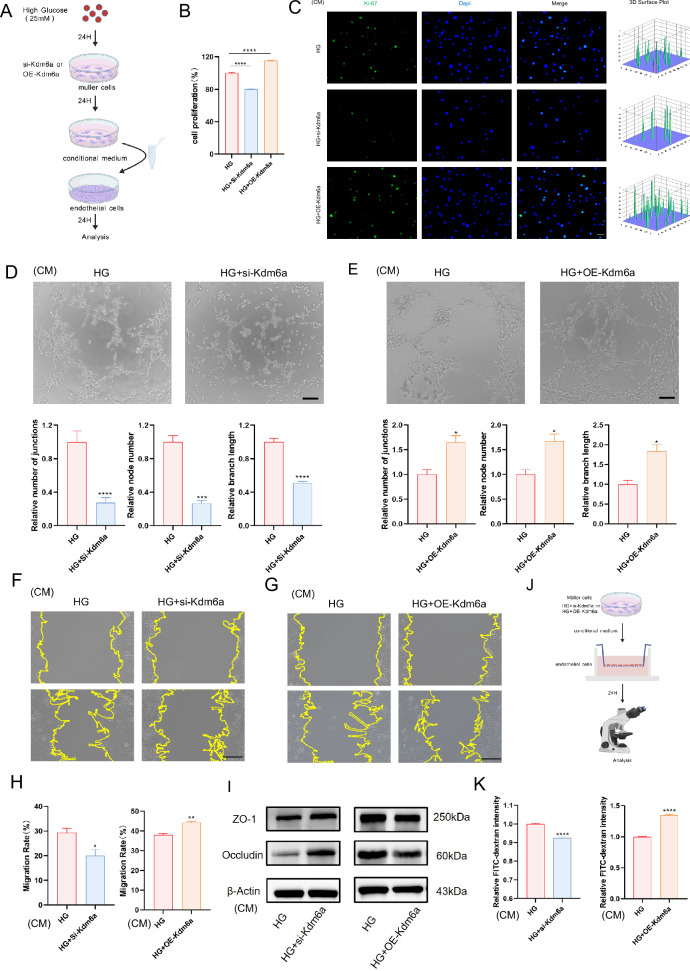
Kdm6a expression in Müller cells modulates endothelial vascularization and barrier integrity in vitro. **A** Schematic diagram of the co-culture system for endothelial cells and Müller cells following Kdm6a knockdown or overexpression. **B** Endothelial cell proliferation was measured by CCK-8 assay under different co-culture conditions (*n* = 6). **C** Ki-67 Immunofluorescence analysis of endothelial cell proliferation in co-culture system (*n* = 3). Scale bar 50 μm. **D** Representative images of in vitro tube formation assays. Endothelial cells co-cultured with Müller cells conditioned under: HG+si-NC or HG+si-Kdm6a, Scale bar 100 μm, *n* = 3. **E** Representative images of in vitro tube formation assays. Endothelial cells co-cultured with Müller cells conditioned under: HG + OE-Vector or HG + OE-Kdm6a, Scale bar:100 μm (*n* = 3). **F**, **G** Wound healing assay of endothelial cells co-cultured with HG-treated Müller cell supernatants following Kdm6a silencing or Kdm6a overexpression (*n* = 3). Scale bar: 100 μm. **H** Quantitative analysis of wound healing capacity in different co-cultured conditions (*n* = 3). **I** Western blot analysis of ZO-1 and Occludin expression in endothelial cells treated with conditioned media from HG-exposed Müller cells (*n* = 3). **J** Schematic Diagram of Endothelial Barrier Integrity Assessment Using FITC-Dextran in the Co-culture System. **K** FITC-dextran permeability assay in endothelial-Müller cell co-culture (*n* = 4). Data are presented as means ± SEM, unpaired two-tailed t-test, *p < 0.05, **p < 0.01, ***p < 0.001, ****p < 0.0001.

To validate whether Kdm6a overexpression in Müller cells may promote endothelial cell proliferation, a co-culture system was established with Müller cells and vascular endothelial cells (Fig. 4A). The mouse retina Müller cells were first exposed to high glucose (HG) stimulation, followed by transfection with either Kdm6a overexpression plasmid or Kdm6a-specific siRNA. The conditioned medium (CM) from these treated Müller cells was then collected and used to treat mouse brain-derived endothelial cells (ECs). Compared to si-NC group, the conditioned medium from si-Kdm6a group of Müller cells suppressed endothelial cell proliferation in CCK-8 assay (Fig. 4B), increased the active marker Ki-67 signal in immunohistochemical assay (Fig. 4C and Supplementary Fig. 4A), reduced tube formation (Fig. 4D), and cell migration (Fig. 4F, H). Conversely, CM from Kdm6a-overexpressing Müller cells exacerbated tube formation (Fig. 4E) and cell migration (Fig. 4G, H). These data suggest that Kdm6a presence in Müller cells under hyperglycemic condition promotes endothelial cell proliferation, migration, and angiogenic activity.

We further investigated whether overexpressing Kdm6a in Müller cells destroys the endothelial barrier function. Our findings demonstrated that downregulation of Kdm6a in Müller cells significantly enhanced the expression of tight junction proteins, including ZO-1 and Occludin, in ECs. Consistent with this, FITC-dextran permeability assays showed that reduced Kdm6a expression in Müller cells resulted in significantly less fluorescent dye leakage, indicating improved endothelial barrier integrity (Fig. 4I−K and Supplementary Fig. 4B, C). These findings collectively demonstrate that Kdm6a presence in Müller cells effectively exacerbates high glucose-induced endothelial dysfunction in vitro.

### High glucose triggers ferroptosis and upregulates Kdm6a expression in Müller cells

**Fig. 5:**
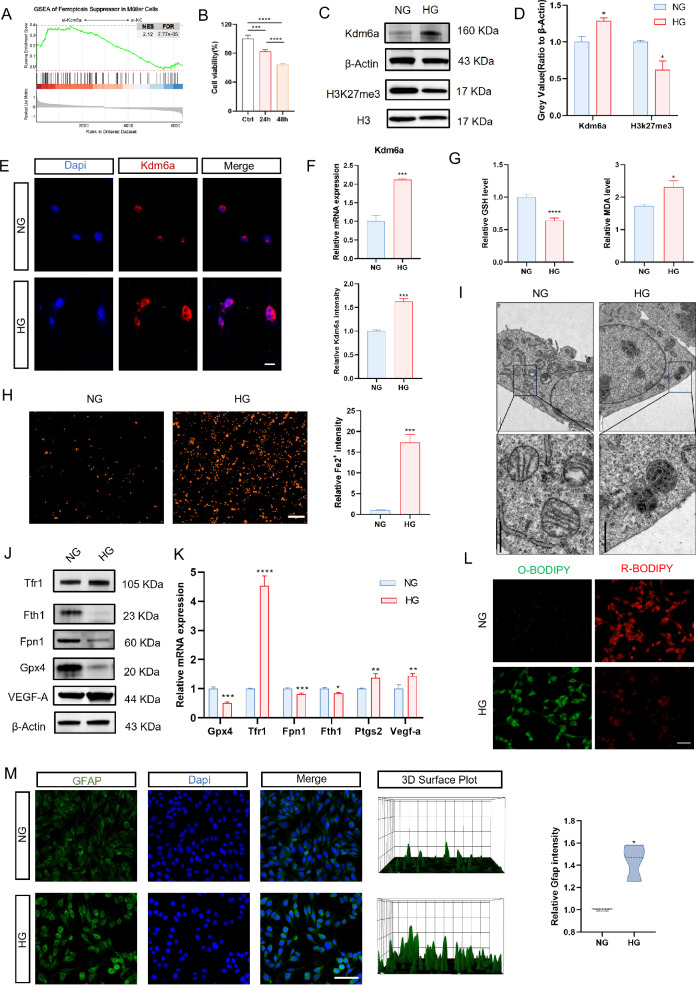
High glucose-induced Kdm6a elevation exacerbates ferroptosis in Müller cells. **A** GSEA using Hallmark gene sets (MSigDB) on scRNA-seq data. Normalized Enrichment Scores (NES) and false discovery rates (FDR) are shown. **B** CCK-8 assay analysis of Müller cell viability under HG conditions (*n* = 6). **C**, **D** The protein level of Kdm6a and the level of H3K27me3 in Müller cells after high glucose stimulation, *n* = 4. **E** Immunofluorescence staining of Kdm6a after HG stimulation or normal condition of Müller cells. Scale bar 50 μm, *n* = 3. **F** The mRNA level of Kdm6a in Müller cells after HG stimulation. *n* = 3, **G** GSH level and MDA level in Müller cells with HG treatment or NG. *n* = 6. **H** FerroOrange (Fe²⁺) staining in high glucose-treated Müller cells versus normal condition Müller cells, Scale bar 200 μm. *n* = 6. **I** TEM analysis of Müller cells under NG and HG conditions. Scale bar:1 μm and 500 nm. **J** The protein level of Tfr1, Fth1, Fpn1, Gpx4, and VEGF-A in Müller cells under NG and HG conditions, *n* = 3. **K** qPCR analysis of Ptgs2, Tfr1, Fth1, Fpn1, Gpx4, and Vegf-α in Müller cells under NG and HG conditions, *n* = 3. **L** Representative fluorescence images of BODIPY staining (green) under NG and HG conditions. Scale bar 50 μm, *n* = 3. **M** Representative immunofluorescence images of GFAP expression in Müller cells from control and high-glucose-treated groups. Scale bar 50 μm. Data shown as mean ± SEM, one-way ANOVA followed by Tukey’s post hoc test and unpaired two-tailed Student’s t-test. *p < 0. 05, **p < 0. 01, ***p < 0.001, ****p < 0.0001.

To establish whether HG directly triggers ferroptosis in Müller cells, we first analyzed ferroptosis-related pathways. Gene Set Enrichment Analysis (GSEA) revealed that reduced Kdm6a expression in Müller cells correlates with elevated transcript levels of ferroptosis-suppressor genes (Fig. 5A) in DR model. Subsequent 30 mM glucose time-course dependently reduced cell viability after 48-h exposure (Fig. 5B and Supplementary Fig. 5A, C). Immunoblotting assay and qPCR analyses demonstrated that HG stimulation markedly upregulated VEGF-A expression in Müller cells (Fig. 5J, K, Supplementary Fig. 5B, D). This was accompanied by increased GFAP immunoreactivity (Fig. 5M). These data suggest that prolonged high glucose challenge induces progressive Müller cell dysfunction.

Consistent with the pathological activation of Müler cells, parallel investigations of epigenetic regulators showed that HG treatment significantly elevated Kdm6a protein and mRNA levels while reducing H3K27me3 modification (Fig. 5C–F). High glucose challenge significantly reduced GSH content and elevated MDA levels compared to normal medium (Fig. 5G). The intensity of FerroOrange probe in HG group is significantly higher than normal group of Müller cells (Fig. 5H). Moreover, BODIPY staining revealed a marked increase in oxidative lipid accumulation in Müller cells under HG conditions, further supporting the induction of lipid peroxidation (Fig. 5L and Supplementary Fig. 5E). To investigate whether HG-induced ferroptosis in Müller cells is associated with ultrastructural mitochondrial damage, we performed transmission electron microscopy (TEM) analysis on Müller cells. Compared to normal medium group, the mitochondria of HG group indicated reduction in mitochondrial volume and a marked disappearance of cristae structures (Fig. 5I and Supplementary Fig. 5F). Specifically, we observed significant downregulation of Gpx4, Fth1, as well as decreased Fpn1 expression and increased Tfr1 levels (Fig. 5J, K and Supplementary Fig. 5B). Notably, treatment with the ferroptosis inhibitor ferrostatin-1 (Fer-1) and Deferoxamine (DFO) significantly rescued high glucose-induced cytotoxicity in Müller cells (Supplementary Fig. 5G, H). These results demonstrate that high glucose induces ferroptosis in Müller cells, characterized by lipid peroxidation, mitochondrial damage, and dysregulation of iron metabolism.

### Kdm6a mediates high glucose-induced ferroptosis and mitochondrial dysfunction

**Fig. 6:**
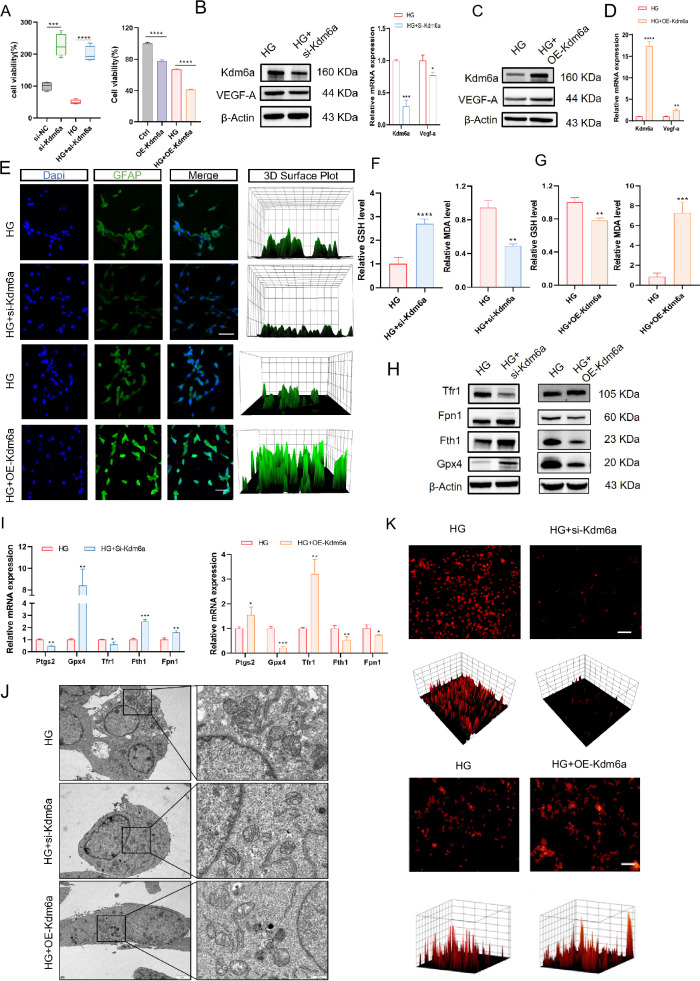
Kdm6a regulates the ferroptosis signaling pathway in Müller cells under high glucose conditions in a bidirectional manner. **A** CCK-8 analysis of Müller cell viability under high glucose conditions following Kdm6a knockdown or overexpression (*n* = 6). **B** Western blots and qPCR of Kdm6a and VEGF-A expression in HG, and HG+si-KDM6A treated Müller cells (*n* = 3). **C** The protein level of Kdm6a and VEGF-A expression in HG, and HG + OE-KDM6A-treated Müller cells (*n* = 3). **D** The mRNA level of Kdm6a and VEGF-A expression in HG and HG + OE-KDM6A-treated Müller cells (*n* = 3). **E** Immunofluorescence Analysis of GFAP expression in Müller Cells under HG conditions (30 mM) with si-Kdm6a or OE-Kmd6a. Scale bar 50 μm, *n* = 6. **F** Intracellular GSH and MDA levels in Müller cells under HG conditions with or without KDM6A knockdown (*n* = 6). **G** Intracellular GSH and MDA levels in Müller cells under HG conditions with or without KDM6A overexpression (*n* = 6). **H** The protein level of Tfr1, Fth1, Fpn1and Gpx4 expression in HG, HG+si-Kdm6a, and HG + OE-Kdm6a treated Müller cells, n = 3. **I** The mRNA level of Ptgs2, Tfr1, Fth1, Fpn1and Gpx4 expression in HG, HG+si-Kdm6a, and HG + OE-Kdm6a treated Müller cells (*n* = 3). **J** TEM Analysis of Müller Cells under HG and HG +si-Kdm6a or HG + OE-Kdm6a. Scale bar: 2 μm and 500 nm, *n* = 3. **K** Representative images of FerroOrange staining in Müller cells. (UP) Comparison between HG and HG+si-Kdm6a groups. (Down) Comparison between HG and HG + OE-Kdm6a groups. Scale bar: 100 μm (*n* = 6). Data are presented as means ± SEM, one-way ANOVA followed by Tukey’s post hoc test and unpaired two-tailed Student’s t-test, *p < 0. 05, **p < 0. 01, ***p < 0.001, ****p < 0.0001.

We next investigated whether Kdm6a mediates ferroptosis in Müller cells. Compared to si-NC group, cell viability of siKdm6a-treated Müller cells significantly attenuated HG-induced reduction of cell viability (Fig. 6A). Conversely, Kdm6a overexpression exacerbated the HG-mediated decline of cell viability (Fig. 6A). Immunoblotting and qPCR analyses demonstrated that Kdm6a knockdown significantly attenuated high glucose-induced VEGF-A upregulation in Müller cells (Fig. 6B and Supplementary Fig. 6A) and Kdm6a overexpression accelerated VEGF-A upregulation (Fig. 6C, D and Supplementary Fig. 6B). This suppression effect was further corroborated by immunofluorescence staining showing a concomitant reduction in GFAP expression (Fig. 6E and Supplementary Fig. 6C). Kdm6a overexpression increased GFAP expression (Fig. 6E and Supplementary Fig. 6D). These data suggest that Kdm6a mediates high glucose challenge induced dysfunction in Müller cell.

We further investigated whether Kdm6a mediates ferroptosis of Müller cells. We observed significantly increased intracellular GSH levels and concurrently reduced MDA content in siKdm6a-treated Müller cells compared to si-NC group, when cells were under high glucose challenge (Fig. 6F). Conversely, the GSH levels in Kdm6a overexpressing Müller cells were significantly lower and MDA content higher than vehicle group (Fig. 6G). As to the analysis on ferroptosis-related markers at both mRNA and protein levels, we found that higher anti-ferroptopic Gpx4, Fth1 and Fpn1 expression in si-Kdm6a treated Müller cells than those of si-NC cells, while lower Tfr1 level (Fig. 6H,I, and Supplementary Fig. 6E). In an opposite regulatory pattern, we observed lower Gpx4, Fth1 and Fpn1 expression along with elevated TFR1 level in Kdm6a-overexpressing cells than those in vehicle group (Fig. 6H, I, and Supplementary Fig. 6F). Consistently, the TEM images showed that siKdm6a partially attenuated HG-induced mitochondrial damage, as evidenced by preserved cristae structure and reduced mitochondrial shrinkage (Fig. 6J and Supplementary Fig. 6G). Overexpressing Kdm6a exacerbated HG-mediated mitochondrial injury, showing more severe cristae disintegration and further reduction in mitochondrial volume (Fig. 6J and Supplementary Fig. 6H). Modulation of Kdm6a expression bidirectionally regulated intracellular Fe²⁺ accumulation, with knockdown decreasing and overexpression increasing Fe²⁺ levels (Fig. 6K and Supplementary Fig. 6I). These findings identify Kdm6a as a critical mediator of high glucose-induced ferroptotic cell death and mitochondrial dysfunction in Müller cells.

### Kdm6a promotes ferroptosis via regulating lipid peroxidation and metabolic genes

**Fig. 7:**
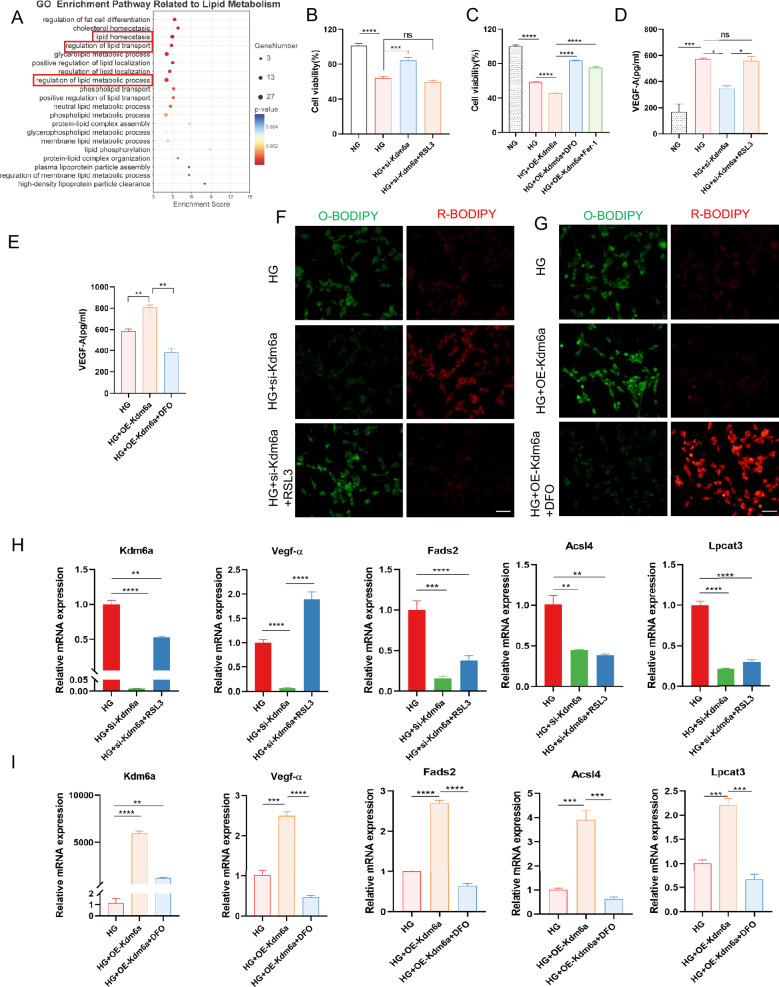
KDM6A regulates lipid metabolism and VEGF-A secretion in Müller cells under high glucose conditions. **A** Lipid metabolism-related GO terms in Müller cells from scRNA-seq. **B** CCK-8 analysis of Müller cell viability under different treatments: NG, HG, HG+si-Kdm6a, HG+si-Kdm6a + RSL3, *n* = 6. **C** CCK-8 analysis of Müller cell viability under different treatments: NG, HG, HG + OE-Kdm6a, HG + OE-Kdm6a+DFO, HG + OE-Kdm6a+Fer-1, *n* = 6. **D** ELISA analysis of VEGF-A concentration in Müller cells culture supernatants. Cells were treated with NG, HG, HG+si-Kdm6a, or HG+si-Kdm6a + RSL3, *n* = 6. **E** VEGF-A concentration in the supernatants of Müller cells measured by ELISA under different treatments: HG, HG + OE-Kdm6a, HG + OE-Kdm6a+DFO, *n* = 6. **F** Representative BODIPY fluorescence images in HG, HG+si-Kdm6a, HG+si-Kdm6a + RSL3 treated Müller cells, *n* = 3. **G** Representative BODIPY fluorescence images in: HG, HG + OE-Kdm6a, HG + OE-Kdm6a+DFO treated Müller cells, *n* = 3. **H** The mRNA level of Kdm6a, Vegf-α, Fads2, Acls4, and Lpcat3 expression in HG, HG+si-Kdm6a, HG+si-Kdm6a + RSL3 treated Müller cells, *n* = 3. **I** The mRNA level of Kdm6a, Vegf-α, Fads2, Acls4, and Lpcat3 expression in HG, HG + OE-Kdm6a, HG + OE-Kdm6a+DFO treated Müller cells, *n* = 3. Data are presented as means ± SEM, one-way ANOVA followed by Tukey’s post hoc test, *p < 0.05, **p < 0. 01, ****p < 0.0001, ***p < 0.001. O-BODIPY oxidized BODIPY, R-BODIPY reduced BODIPY.

Given the critical role of lipid metabolic reprogramming in modulating ferroptosis sensitivity, we next investigated whether Kdm6a influences lipid metabolic pathways in Müller cells. GO enrichment analysis of scRNA-seq data revealed that Kdm6a knockdown induced significant remodeling of lipid metabolism (Fig. 7A). Functionally, silencing Kdm6a markedly improved the viability of HG-treated Müller cells, and this protective effect was abrogated by the ferroptosis inducer RSL3. Consistent with this, under NG conditions, Kdm6a knockdown attenuated RSL3-induced cytotoxicity, further confirming that Kdm6a depletion enhances cellular resistance to ferroptosis (Fig. 7B and Supplementary Fig. 7A). Conversely, the reduction in cell viability resulting from Kdm6a overexpression was effectively reversed by treatment with the ferroptosis inhibitors DFO or Fer-1 (Fig. 7C).

Notably, under NG conditions, Kdm6a knockdown significantly improved cell viability and inhibited RSL3-induced VEGF-A upregulation, confirming its intrinsic anti-ferroptotic role (Supplementary Fig. 7A, B). Consistently, Kdm6a knockdown attenuated HG-induced VEGF-A upregulation at both mRNA and protein levels, and this suppressive effect was abrogated by RSL3 treatment (Fig. 7D, H), whereas DFO mitigated the Kdm6a overexpression-induced upregulation of VEGF-A (Fig. 7E, I). These findings were further supported by lipid peroxidation assays: RSL3 reversed the reduction in oxidative lipid accumulation caused by Kdm6a silencing under HG, as evidenced by increased O-BODIPY staining (Fig. 7F and Supplementary Fig. 7C). In contrast, DFO attenuated the elevated O-BODIPY signal in Kdm6a-overexpressing cells (Fig. 7G and Supplementary Fig. 7D), confirming the role of Kdm6a in promoting lipid peroxidation.

To explore the molecular basis through which Kdm6a regulates lipid metabolism during ferroptosis, we assessed the expression of key enzymes involved in lipid peroxidation. Under HG conditions, Kdm6a knockdown significantly downregulated the mRNA levels of Acsl4, Lpcat3, and Fads2 (Fig. 7H), genes critical for polyunsaturated fatty acid (PUFA) synthesis and membrane lipid remodeling. Notably, RSL3 treatment abrogated these downregulatory effects. Conversely, Kdm6a overexpression upregulated these genes, an effect that was partially reversed by DFO treatment (Fig. 7I). Together, these data further support the role of Kdm6a in promoting ferroptosis through modulating lipid peroxidation-related gene expression.

### Kdm6a directly regulates ferroptosis pathway through H3K27me3

**Fig. 8:**
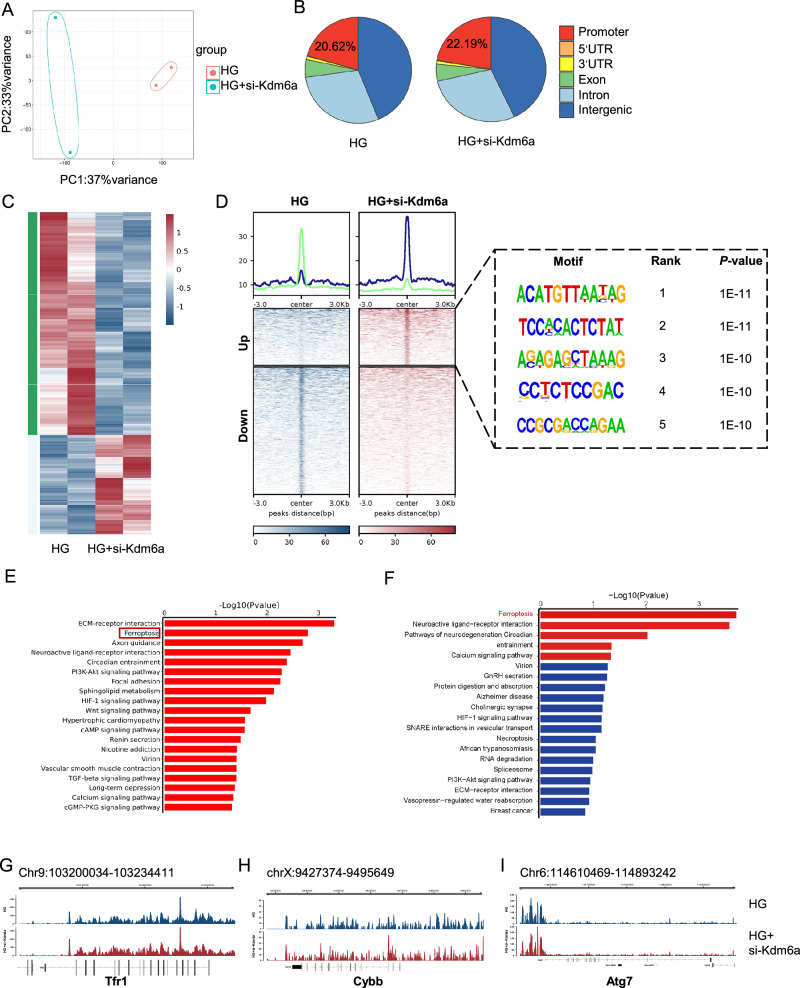
CUT&Tag profiling identifies direct epigenetic regulation of ferroptosis-related genes by Kdm6a via H3K27me3 demethylation. **A** PCA analysis of the targeted H3K27me3 peaks of the Müller cell under HG (30 mM) with or without Kdm6a knockdown. *n* = 2 biological replicates. **B** Distribution of H3K27me3 peaks on the functional regions of genes. **C** Heatmap analysis of differential H3K27me3 peaks in Müller cells under HG (30 mM) with or without Kdm6a knockdown. **D** Metaplot and heatmap showing H3K27me3 enrichment of individual genes in Müller cells under HG (30 mM) with or without Kdm6a knockdown. **E** KEGG analysis of significantly enriched pathways in the gene sets mapped to the all differentially expressed H3K27me3 peak in Müller cells under HG (30 mM) with or without Kdm6a knockdown. **F** KEGG analysis of significantly enriched pathways in the gene sets mapped to the upregulated H3K27me3 peak in Müller cells under HG (30 mM) with or without Kdm6a knockdown. **G**–**I** Genome browser view showing H3K27me3 enrichment near Tfr1, Cybb, and Atg7 in Müller cells under HG (30 mM) with or without Kdm6a knockdown.

To investigate the molecular mechanism by which Kdm6a regulates the ferroptosis pathway, we performed CUT&Tag assays with an anti-H3K27me3 antibody in Müller cells under high glucose challenge. This approach allowed us to precisely map the genome-wide distribution of H3K27me3 modifications between siNC and siKdm6a-treated cells. Principal component analysis (PCA) of H3K27me3-targeted peaks revealed distinct clustering patterns, with clear separation between the HG+si-Kdm6a and HG treatment groups (Fig. 8A). Analysis of promoter-associated H3K27me3 peaks revealed that depletion of Kdm6a led to a marked elevation of H3K27me3 deposition at promoter regions, with peak coverage increasing from 20.62% in HG cells to 22.19% in HG+si-Kdm6a cells (Fig. 8B). Genome-wide profiling identified significant H3K27me3 redistribution following Kdm6a knockdown, with 1276 genes (69.16%) exhibiting decreased H3K27me3 peak intensity and 569 genes (30.84%) showing increased peaks among the 1,845 significantly altered loci (Fig. 8C). Analysis of H3K27me3 peak distribution revealed predominant enrichment near transcription start sites (TSS) in both experimental groups (Fig. 8D). HOMER motif analysis of regions showing increased H3K27me3 enrichment in CUT&Tag data identified five significantly enriched DNA sequence motifs (Fig. 8D). Besides, KEGG pathway analysis revealed that genes exhibiting increased H3K27me3 peaks following Kdm6a knockdown were significantly enriched in the ferroptosis pathway (Fig. 8E, F). Analysis of ferroptosis-related genes indicated an increase in H3K27me3 peak intensity at the promoter region of Tfr1, Cybb, and Atg7 following Kdm6a knockdown (Fig. 8G–I). These findings demonstrate that KDM6A directly promotes the ferroptosis pathway through H3K27me3-mediated epigenetic regulation.

## Discussion

In this study, we investigated the role of Kdm6a in mediating vascular dysfunction of diabetic retina. We found that silencing Kdm6a effectively suppressed aberrant vascular dysfunction, reducing pathological neovascularization, vascular leakage, and structural disarray in the diabetic retina. Single-cell analysis revealed that Kdm6a knockdown in Müller cells specifically reduced retinal angiogenesis by attenuating VEGF-A production and enhancing intercellular signaling with endothelial cells. Conversely, overexpressing Kdm6a in Müller cells exacerbated vascular dysfunction, promoting endothelial cell proliferation and enhancing VEGF-A production. High glucose exposure increased Kdm6a expression in Müller cells, leading to ferroptosis. Mechanistically, Kdm6a promoted ferroptosis by decreasing H3K27me3 deposition at the promoter regions of ferroptosis-related genes, upregulating pro-ferroptotic genes such as Tfr1. These findings highlight Kdm6a as a critical regulator of both angiogenesis and ferroptosis in Müller cells, contributing to the pathogenesis of diabetic retinopathy.

We revealed that Kdm6a plays a central role in modulating ferroptosis, a form of regulated cell death characterized by lipid peroxidation and iron accumulation. Single-cell sequencing and molecular analyses demonstrated that high glucose stimulation upregulates Kdm6a expression in Müller cells, leading to increased oxidative stress, lipid peroxidation, and mitochondrial damage. Importantly, Kdm6a knockdown attenuates these effects, as evidenced by reduced MDA levels, increased GSH content, and preserved mitochondrial structure. Our results from single-cell data mining, gene expression profiling, and fluorescent imaging collectively demonstrate that KDM6A promotes ferroptosis in Müller cells by modulating lipid remodeling, consistent with the mechanism reported in the literature [14]. Furthermore, our CUT&Tag assays revealed that Kdm6a directly regulates the ferroptosis pathway through H3K27me3-mediated epigenetic regulation. Specifically, Kdm6a knockdown leads to increased H3K27me3 deposition at promoter regions of ferroptosis-related genes, such as Tfr1, thereby suppressing ferroptosis. Our findings suggest that Kdm6a acts as a positive regulator of ferroptosis in Müller cells under high glucose conditions, contributing to their pathological activation and subsequent retinal vascular dysfunction.

Currently, the mechanisms and methods for selectively regulating ferroptosis remain elusive, requiring selective activation or inhibition of target genes in specific tissues, cells, and/or disease environments to combat ferroptosis [27]. Müller cells have more active metabolism and higher ROS levels, making them more susceptible to oxidative stress-induced ferroptosis [28]. In addition, studies have shown that Müller cells typically require a large amount of iron, which may further make these cells sensitive to iron induced cell death. Müller cells may also counteract these metabolic and oxidative loads through additional genetic or epigenetic mechanisms to reduce their susceptibility to ferroptosis, such as inhibiting Keap1 expression or upregulating Nrf2 [29]. Therefore, selecting appropriate persistent targeting methods for Müller cells to control ferroptosis is highly attractive for restoring Müller cell function in DR and may provide a therapeutic safety window. There are multiple methods to enhance the specific regulation of iron death in Müller cells under different conditions, such as through drug delivery carriers, optimizing their biological distribution and pharmacokinetics, and selecting targets and mechanisms.

Crucially, our findings demonstrate that the therapeutic benefits of Kdm6a knockdown extend beyond vasoprotection to encompass significant neuroprotective effects. Diabetic retinopathy is increasingly recognized as a neurovascular disease, where neuronal dysfunction and neurodegeneration occur early and may precede clinically evident microvascular pathology [30]. In this study, the preserved retinal thickness observed following Kdm6a silencing serves as direct morphological evidence of neuroprotection, since reductions in retinal thickness, especially of the inner retinal layers, are now considered reliable structural biomarkers of diabetic retinal neurodegeneration [31]. By rescuing Müller cells from ferroptosis, Kdm6a knockdown likely preserves their critical homeostatic functions. Müller cells are the principal glia in the retina and maintain neuronal survival by clearing extracellular glutamate via GLAST/EAAT1 and converting it to glutamine through glutamine synthetase, thereby preventing excitotoxicity [32], and by secreting neurotrophic factors such as ciliary neurotrophic factor, glial cell line-derived neurotrophic factor, and pigment epithelium-derived factor. This neuroprotective effect complements the suppression of angiogenesis and vascular leakage, addressing both the neural and vascular components of DR. Consequently, targeting the Kdm6a-mediated ferroptosis pathway offers a comprehensive therapeutic strategy that simultaneously safeguards retinal structural integrity and vascular stability. While our study provides strong evidence for the role of Kdm6a in DR, several limitations and future directions warrant consideration. First, the mechanisms by which Kdm6a regulates VEGF-A expression in Müller cells remain to be fully elucidated. Second, the long-term effects of Kdm6a knockdown on retinal function and visual outcomes in diabetic mice need to be investigated. Third, the potential off-target effects of Kdm6a inhibition in other retinal cell types should be carefully evaluated. Finally, translational studies are needed to assess the safety and efficacy of Kdm6a-targeted therapies in human clinical trials.

In summary, Kdm6a promotes ferroptosis in Müller cells, leading to angiogenesis, endothelial barrier function, and retinal vascular abnormalities in DR. Our findings provide a foundation for further exploration of Kdm6a as a key target of retinal vascular homeostasis in DR.

## Methods and materials

### Animal experiment

All animal experiments were performed in compliance with the ARVO Statement for the Use of Animals in Ophthalmic and Vision Research. The study protocol was reviewed and approved by the Animal Ethics Committee of the Eye & Ear, Nose, and Throat Hospital of Fudan University. The db/db (15 weeks old) mice C57BL/6 (6–8 weeks old) were obtained from Shanghai Legen Biotechnology Co., Ltd (Shanghai, China), while the Kdm6a^ki/ki^ and Kdm6a^ki/y^ mice were bred and maintained as previously reported [33]. Mice were kept in a 12 h light/dark cycle and 10% humidity at 20 ± 4 °C. They were given free access to chow and water.

### Patient samples

This study was reviewed and approved by the Ethical Committee of the Eye & Ear, Nose, and Throat Hospital of Fudan University, Shanghai, China (no: 2025203). All participants provided written informed consent for both surgical procedures and research participation. The proliferative membrane samples were obtained from patients undergoing vitrectomy with PDR and idiopathic epiretinal membranes (ERM) (screened negative for diabetes mellitus history). Immediately following vitrectomy, the excised proliferative membranes were placed in pre-chilled 4 °C containers and, within 15 min of collection, were flash-frozen in liquid nitrogen for long-term storage at −80 °C in cryopreservation tubes to ensure optimal preservation of tissue integrity for subsequent molecular analyses.

### Diabetic mice model

Kdm6a^ki/ki^ or Kdm6a^ki/y^ mice (6–8 weeks old, body weight approximately 25 g) were randomly assigned to control and experimental groups. Type 1 diabetes was induced through intraperitoneal injection of streptozotocin (STZ, MaoKangbio, China) as follows: Mice were fasted for 12 h prior to the initial injection and subsequently received daily STZ injections (55 mg/kg/day, dissolved in 0.1 M sodium citrate buffer, pH 4.5) for 5 consecutive days. The control group received equivalent volumes of sodium citrate buffer alone. Diabetes was confirmed by measuring fasting blood glucose levels 72 h after the final injection using a OneTouch glucose meter. Mice with blood glucose levels exceeding 300 mg/dL on two consecutive measurements were classified as diabetic and included in subsequent experiments. The mice were sacrificed by neck dislocation. The retina from each mouse was collected, frozen with liquid nitrogen, and stored at −80 °C.

### Intravitreal injection in diabetic mouse models

During the experimental procedure, mice were deeply anesthetized and maintained on a thermostatically controlled heating pad to ensure stable body temperature. A Hamilton syringe was carefully inserted into the vitreous cavity at a 45° angle relative to the posterior corneal edge and stabilized for 30 s prior to injection. *Kdm6a*^*ki/ki*^ and *Kdm6a*^*ki/y*^ mice received 1.5 μL of AAV-shH10Y or AAV-shH10Y-Cre (HanBio, China), respectively. Similarly, db/db mice were administered 1.5 μL of either si-NC (HanBio, China) or si-Kdm6a (HanBio, China). Following intravitreal injections, antibiotic ointment was applied to the ocular surface to prevent corneal desiccation and mitigate the risk of intraocular infection.

### In vivo treatment with GSK-J4 in diet-induced obese mice

Sixteen-week-old male diet-induced obese mice (DIO, Gempharmatech Co, China) were used in this study. GSK-J4 (Sigma-Aldrich, USA) was dissolved in 2% DMSO and administered intraperitoneally at a dose of 30 mg/kg once daily for 14 days. Control mice received vehicle treatment (2% DMSO). After the final administration, mice were euthanized by cervical dislocation, and eyes were enucleated and fixed in Davidson’s Fixative for subsequent analysis.

### Single-cell library preparation and sequencing

The eyeballs were obtained from db/db mice, which underwent different intraocular injection treatments, specifically siRNA targeting Kdm6a (si-Kdm6a) and its corresponding negative control (si-NC). Retinas were immediately dissected in ice-cold 1×PBS and mechanically minced into small fragments (<1 mm³) on a chilled surface. Retinas were dissected in precooled PBS and thoroughly minced on ice. Each retina was incubated with 1 ml digestion buffer (700 μL reagent grade water, 100 μL of freshly prepared 50 mM L-Cysteine (Sigma), 100 μL of 10 mM EDTA, 10 μL of 60 mM 2-mercaptoethanol (Sigma), and 1 mg/mL papain (Worthington)) for 30 min at 37 °C. Cell count and viability were assessed, and only the sample with a viability exceeded 90%.

The 10x Genomics platform employed microfluidic technology to encapsulate beads bearing cell-specific barcodes with individual cells in aqueous droplets. Following the collection of cell-containing droplets, the cells were lysed in situ. Single-cell GEMs were generated by hybridizing cellular mRNA molecules to the barcoded oligonucleotides on the bead surface. Reverse transcription was performed to synthesize cDNA libraries, and an indexed sequencing adapter was incorporated during library preparation, which permitted the assignment of each read to its sample of origin.

### Single-cell sequencing data analysis

The raw sequencing data were demultiplexed to generate FASTQ files, which were subsequently aligned to the mouse reference genome using Cell Ranger (version 7.0.1, 10x Genomics, USA). The Seurat package (v5.2.1) in R (v4.4.2) was used to integrate and process the data [34]. Following scRNA-seq, we performed quality control and batch correction. Cells were retained if they satisfied all the following criteria: 200-7000 features, RNA counts between 1000 and the 97th percentile, log10GenesPerUMI > 0.7, and <3% hemoglobin content. The mitochondrial gene expression below 15% were kept because the retinas are highly energy-consuming tissues [34] (Supplementary Fig. 2A).

After log-normalization, scaling, and identification of highly variable genes, PCA was carried out, and the first 20 principal components were embedded in two-dimensional space with UMAP for visualization. Clusters were annotated with canonical markers, and retinal-pigment-epithelium or unclassified clusters were excluded from downstream analyses. Intercellular communication networks were reconstructed with the CellChat R package [35]. Functional enrichment analyses were performed with KEGG pathways and GO terms, and ferroptosis involvement was evaluated using gene sets curated in FerrDb V2 [36].

Endothelial cells underwent further subclustering based on markers derived from previous studies [23, 37]. The proportions of endothelial cell subpopulations between the two experimental groups were then compared. Additionally, gene sets corresponding to upregulated genes of tip and stalk cells identified from the literature were utilized to calculate module scores with the Add Module Score function in Seurat. The raw sequence data reported in this paper have been deposited in the Genome Sequence Archive (Genomics, Proteomics & Bioinformatics 2021) in the National Genomics Data Center (Nucleic Acids Res 2022), China National Center for Bioinformation/Beijing Institute of Genomics, Chinese Academy of Sciences (GSA: CRA028968), which are publicly accessible at https://ngdc.cncb.ac.cn/gsa.

### Bioinformatics analysis of public RNA-seq data

Raw RNA-seq datasets (GSE160306) were retrieved from the GEO database. Gene expression differences were analyzed using the limma package (version 3.62) in R (version 4.4.3). Comparisons were performed between Ctrl versus PDR and NPDR versus PDR. Statistical significance thresholds were set at |log2(fold change)| > 0.5 with adjusted *p* value < 0.05 (Benjamini-Hochberg multiple testing). Data visualization was performed using the ggplot2 package (v3.5.2) in R (v4.4.3).

### Cell culture and treatments

We incubated the immortalized murine müller glia cell line QMMuC-1 (RRID:CVCL_UW39, BLUEFBIO, China) [38], and mouse brain microvascular endothelial cells bEnd.3 (RRID:CVCL_0170,WHELAB, China)was cultured in DMEM supplemented with 10% FBS and 1% penicillin/streptomycin and maintained at 37 °C in a humidified incubator with 5% CO_2_. All cell lines were authenticated by the suppliers prior to shipment and confirmed to be free of mycoplasma contamination through routine testing. To establish an in vitro model of HG-induced cellular injury, QMMuC-1 cells were cultured in medium supplemented with a high concentration of D-glucose (30.0 mM, Thermo Fisher) for 48 h. The control group was maintained in normal glucose medium containing 5.0 mM D-glucose for the same duration.

### Conditional medium

Müller cells were first treated with high glucose (30 mM) for 24 h, followed by genetic manipulation (Kdm6a overexpression or knockout) and an additional 24-h culture period under the same HG conditions. The culture supernatant was then collected and processed by centrifugation at 300 × *g* for 10 min at room temperature to remove cellular debris, followed by sterile filtration through 0.22 μm filters (Millipore, USA) to generate conditioned medium (CM), which was either used immediately or aliquoted and stored at −80 °C for subsequent experiments.

### siRNA transfection

The negative control (NC) siRNA, Kdm6a-specific targeting siRNA (CUA CGA AUC UCU AAU CUU A dTdT) were produced by HanBio (China). Müller cells were transfected with 50 nM Kdm6a-targeting siRNA (or scrambled control siRNA) using Lipofectamine 2000 (Thermo Fisher Scientific) according to the manufacturer’s instructions. The siRNAs were modified at the 5′ end with cholesterol to improve the absorption by Müller cells and 2-*o*-methylation at the 3′ end to be protected from degradation. Following 12 h of transfection, successful Kdm6a knockdown was confirmed by both qPCR (for mRNA levels) and Western blot (for protein expression). Subsequently, transfected cells were exposed to high glucose (HG) conditions for 24 h under either KDM6A knockdown or control conditions.

### Cleavage under targets and tagmentation (CUT&Tag)

The CUT&Tag assay was performed following an established protocol [39] with minor modifications. Library quality was assessed using an Agilent TapeStation4200 system (Agilent Technologies) to verify size distribution and concentration. Qualified libraries were pooled in equimolar ratios and subjected to 150-bp paired-end sequencing on an Illumina NovaSeq6000 platform. All experimental procedures, including library preparation and primary data analysis, were conducted by Jiayin Biotechnology Ltd. (Shanghai, China).

### Haematoxylin and eosin (HE) staining

At the end of the treatment period, the eyeballs were dissected and placed in special fixation solution (Servicebio, China) overnight at 4 °C. Eyeballs were processed for paraffin embedding and sectioned for histological examination. The Paraffin-embedded tissue sections (4 μm thickness) were first dewaxed in xylene and then rehydrated through a graded series of ethanol solutions. The sections were sequentially stained with hematoxylin and eosin. The images were captured with a Pannoramic Scanner (Pannoramic DESK, Hungary). All antibodies used are listed in Supplementary Table 2.

### Immunohistochemistry

Following dewaxing, gradient rehydration, and high-temperature antigen retrieval, retinal sections were sequentially treated with 3% hydrogen peroxide solution for 15 min to block endogenous peroxidase activity, followed by incubation with 10% goat serum (Sigma, USA) for 30 min to prevent nonspecific binding. The sections were then incubated overnight at 4 °C with a primary antibody against GFAP (1:200 dilution, Abcam). After thorough washing, the sections were treated with an HRP-conjugated secondary antibody (Solarbio, China) for 1 h at room temperature. Immunoreactivity was visualized using a diaminobenzidine (DAB) kit (Solarbio, China), and cell nuclei were counterstained with hematoxylin. The images were captured with a Pannoramic Scanner (Pannoramic DESK, Hungary). All antibodies used are listed in Supplementary Table 2.

### Immunofluorescence staining

bEnd.3 cells (mouse brain microvascular endothelial cells) were cultured and seeded in 12-well plates. Cells were fixed with 4% paraformaldehyde (Solarbio, China) at room temperature for 30 min, permeabilized with 0.1% Triton X-100 (Beyotime Biotechnology, China) for 30 min, and blocked with 10% goat serum (Thermo Fisher Scientific, USA) for 1 h. The cells were then incubated overnight at 4 °C with a primary antibody against KI-67 (1:200 dilution, Thermo Fisher Scientific, USA). The following day, cells were incubated with a FITC-conjugated secondary antibody (CST, USA) for 1 h at room temperature, followed by nuclear counterstaining with DAPI (Beyotime Biotechnology, China). Fluorescence images were captured using a fluorescence microscope (Zeiss imager M2, Germany).

Briefly, paraffin-embedded mouse eye sections were dewaxed in xylene and rehydrated through a graded ethanol series. Antigen retrieval was performed in heated citrate buffer (10 mM, pH 6.0) using a microwave method. Sections were permeabilized with 0.3% Triton X-100 and blocked with 10% goat serum (Sigma, USA). The sections were then incubated overnight at 4 °C with primary antibodies against either GFAP (1:200, Abcam) or CD31 (1:200, Proteintech). After washing, the sections were incubated with Alexa Fluor 488 or T Alexa Fluor 647 secondary antibodies (1:200, Abcam) for 1 h at room temperature, along with nuclear counterstaining using DAPI (Beyotime, China). Fluorescence images were captured using a fluorescence microscope (Zeiss imager M2, Germany). All antibodies used are listed in Supplementary Table 2.

### Retinal trypsin digestion

Eyes were fixed in 4% paraformaldehyde for 48 h, after which retinas were dissected and rinsed overnight in deionized water. The retinas were then incubated with 3% trypsin (Thermo Fisher Scientific, USA) at 37 °C for 3 h with gentle agitation to isolate the vascular network from surrounding tissue. Following digestion, the vessels were stained with periodic acid-Schiff (Solarbio, China) reagent and examined under an optical microscope (Zeiss imager M2, Germany) to assess pathological changes in the retinal vascular architecture.

### Evans blue staining of whole-mount retinas

Mice were anesthetized via intraperitoneal injection of ketamine/xylazine (100 mg/kg and 10 mg/kg body weight, respectively). Evans Blue dye (Yeasen, China; 10 mg/mouse dissolved in 0.2 mL of 0.01 M PBS) was administered through retro-orbital venous sinus injection using established techniques [40]. Ten minutes post-injection, animals were euthanized by pentobarbital overdose (150 mg/kg, i.p.), and eyes were immediately enucleated. Eyeballs were fixed in 4% paraformaldehyde (PFA) for 1 h at room temperature under light-protected conditions. Following fixation, retinas were carefully dissected and flat-mounted on microscope slides. Retinal vascular permeability was assessed using a Zeiss M2 microscope (Germany) with a 10× objective lens. All images were acquired and processed using Zeiss Zen software (version 3.0)

### Western blotting

Retinal tissues or cultured cells were lysed in RIPA buffer supplemented with protease and phosphatase inhibitor cocktail (Beyotime Biotechnology, China). Protein concentrations were quantified using a BCA assay kit (Epizyme Biotechnology, China) according to the manufacturer’s protocol. Equal amounts of protein (20–50 μg) were separated by SDS-PAGE and subsequently transferred onto PVDF membranes (Millipore, USA). Membranes were blocked with QuickBlock™ blocking buffer (Beyotime Biotechnology, China) for 30 min at room temperature, followed by incubation with primary antibodies overnight at 4 °C. After washing, membranes were probed with appropriate HRP-conjugated secondary antibodies for 1 h at room temperature. Protein bands were visualized using enhanced chemiluminescence (ECL) substrate (MCE, USA). Band intensity was quantified using ImageJ software (NIH, USA) with normalization to loading controls. Detailed information regarding primary antibodies, including catalog numbers and dilutions, is provided in Supplementary Table 2.

### Tube formation assay

Matrigel matrix (D1 Medical Technology, China) was thawed overnight at 4 °C. Matrigel (50 μL/well) was pipetted into precooled 96-well plates and allowed to polymerize for 30 min at 37 °C in a humidified5% CO2 incubator. bEnd.3 cells (2 × 10^4^ cells/well) were then seeded onto the polymerized Matrigel surface and cultured under standard conditions (37 °C, 5% CO_2_). After 4–6 h of incubation, capillary-like tube structures were examined and imaged using an optical microscope (Zeiss imager M2, Germany) equipped with a 10× objective. Three random fields per well were captured for quantitative analysis of tube formation.

### Scratch wound healing assay

bEnd.3 cells were seeded in 6-well plates and allowed to adhere overnight. Following experimental interventions, a standardized wound was created in each well using a sterile 10 μL pipette tip. The wells were gently washed three times with PBS to remove dislodged cells and debris, followed by replenishment with conditional medium. Cell migration was monitored at 0 h (immediately post-scratch) and 24 h using a Zeiss imager M2 microscope (Zeiss, Germany) equipped with a 10× objective. Three representative fields per well were captured for quantitative analysis. Wound closure was quantified by measuring the remaining scratch area at 24 h relative to the initial wound area (0 h) using ImageJ software (NIH, USA).

### RNA isolation and quantitative PCR assays

RNA was extracted from cells with TRIzol reagent (Invitrogen™, United States). Then, 2 μg of each RNA sample was transcribed to cDNA using a Reverse Transcription System (Takara, Japan) according to the manufacturer’s instructions. qRT-PCR was performed using TB Green® Premix Ex Taq™ II (Takara, Japan). The thermal cycling conditions were 95 °C for 30 s followed by 40 cycles of 95 °C for 5 s and 60 °C for 34 s. The mRNA expression level was normalized to β-actin, and the 2^−ΔΔCt^ method was used for analysis. The primer sequences are presented in Supplementary Table 1.

### CCK-8 cell viability/proliferation assay

A Cell Counting Kit-8 (CCK-8) (Dojindo, Japan) was used to determine cell viability. Cells (1 × 10^4^) were cultured in a 96-well plate and were pretreated with relevant conditioned medium or HG 30 mM stimulation. After that, cells were incubated with CCK-8 for 1 h at 37 °C, and the absorbance value was measured at 450 nm.

### Malondialdehyde (MDA) assay

The intracellular Malondialdehyde (MDA) level was measured with a Lipid Peroxidation MDA Assay Kit (Solarbio, China) according to the manufacturer’s instructions. The value was normalized to the protein concentration detected by a Pierce BCA protein assay kit (Epizyme Biotechnology, China).

### Measurement of VEGF-A secretion in Müller cells

After the indicated treatments, Müller cells were harvested, and the supernatant was collected. VEGF-A levels in the supernatant were measured using a commercial ELISA kit (R&D Systems, USA) according to the manufacturer’s instructions. Briefly, samples were added to pre-coated 96-well plates, followed by incubation with detection antibody and streptavidin-HRP. After washing, the color reaction was developed with TMB substrate, and the absorbance was measured at 450 nm using a microplate reader. VEGF-A concentrations were calculated from a standard curve and normalized to total protein content (determined by BCA assay).

### GSH assay

The intracellular Glutathione (GSH) level was measured with a Glutathione Assay Kit (Solarbio, China) according to the manufacturer’s instructions. Müller cells in six-well plates were treated with or without HG (30 mM) for 48 h. The absorbance at 412 nm was measured and used to calculate the GSH amount in reference to a GSH standard curve.

### Determination of intracellular free Fe^2+^ level

Müller cells were seeded in 12-well plates and divided into four experimental groups: (1) control (Ctrl), (2) high glucose (HG), (3) HG + Kdm6a knockdown (si-Kdm6a), and (4) HG + Kdm6a overexpression (OE-Kdm6a). Following respective treatments, cells were incubated with 10 μM FerroOrange (Dojindo Molecular Technologies, Japan) at 37 °C for 30 min in the dark. After incubation, cells were washed three times with PBS to remove excess probe. Fluorescent images were acquired using a Zeiss imager M2 microscope with excitation/emission set at 543/580 nm. Quantitative analysis of Fe²⁺ levels was performed by measuring fluorescence intensity using ImageJ software (NIH, USA), with at least three random fields captured per well.

### Lipid ROS detection

Lipid ROS was detected using the BODIPY 581/591 C11 kit (Thermo Fisher Scientific, Waltham, MA, USA). Müller cells were seeded in 6-well plates at a density of 5 × 10⁴ cells/well and treated under various conditions. After treatment, cells were stained with 2 μM C11-BODIPY (581/591) probe for 30 min at 37 °C according to the manufacturer’s protocol. Fluorescence imaging was performed using a fluorescence microscope (Zeiss M2), and images were analyzed using ImageJ software (NIH, Bethesda, MD, USA). Oxidized BODIPY (O-BODIPY) was visualized at 488/510 nm, while reduced BODIPY (R-BODIPY) was detected at 581/591 nm.

### TEM analysis of the mitochondrial ultrastructure

After treatment, Müller cells were fixed with 2.5% glutaraldehyde in 0.1 M phosphate buffer (pH 7.4) for 4 h at 4 °C, followed by two washes with PBS. The cells were then post-fixed with 1% osmium tetroxide for 1 h. Subsequently, samples were dehydrated through a graded ethanol series. Samples were embedded in epoxy resin, followed by slicing. The mitochondrial structure was observed by TEM.

### Statistical analysis

All quantitative data were analyzed blindly using GraphPad Prism 8.0 (GraphPad Software, USA) and are presented as mean ± SEM. For comparisons between two groups, statistical significance was assessed using an unpaired two-tailed Student’s t-test. For comparisons involving three or more groups, one-way ANOVA followed by Tukey’s post hoc test was performed. A *p* value < 0.05 was considered statistically significant. Sample sizes were determined based on previous publications and preliminary data.

## Supplementary information

Supplementary Figure 1

Supplementary Figure 2

Supplementary Figure 3

Supplementary Figure 4

Supplementary Figure 5

Supplementary Figure 6

Supplementary Figure 7

Supplementary Material

Full Scans of Western Blots

## Data Availability

The data supporting the findings of this study are available from the corresponding author upon reasonable request. The raw sequence data reported in this paper have been deposited in the Genome Sequence Archive (Genomics, Proteomics & Bioinformatics 2021) in the National Genomics Data Center (Nucleic Acids Res 2022), China National Center for Bioinformation/Beijing Institute of Genomics, Chinese Academy of Sciences (GSA: CRA028968), which are publicly accessible at https://ngdc.cncb.ac.cn/gsa.
