# Supplementary figures and images for: KDM6A promotes diabetic retinopathy via H3K27me3-dependent ferroptosis in Müller cells
Source: Cell Death Dis. 2026 Apr 29;17(1):571. doi: 10.1038/s41419-026-08816-9 (PMC13265779; doi:10.1038/s41419-026-08816-9)

# Supplementary Figure 1

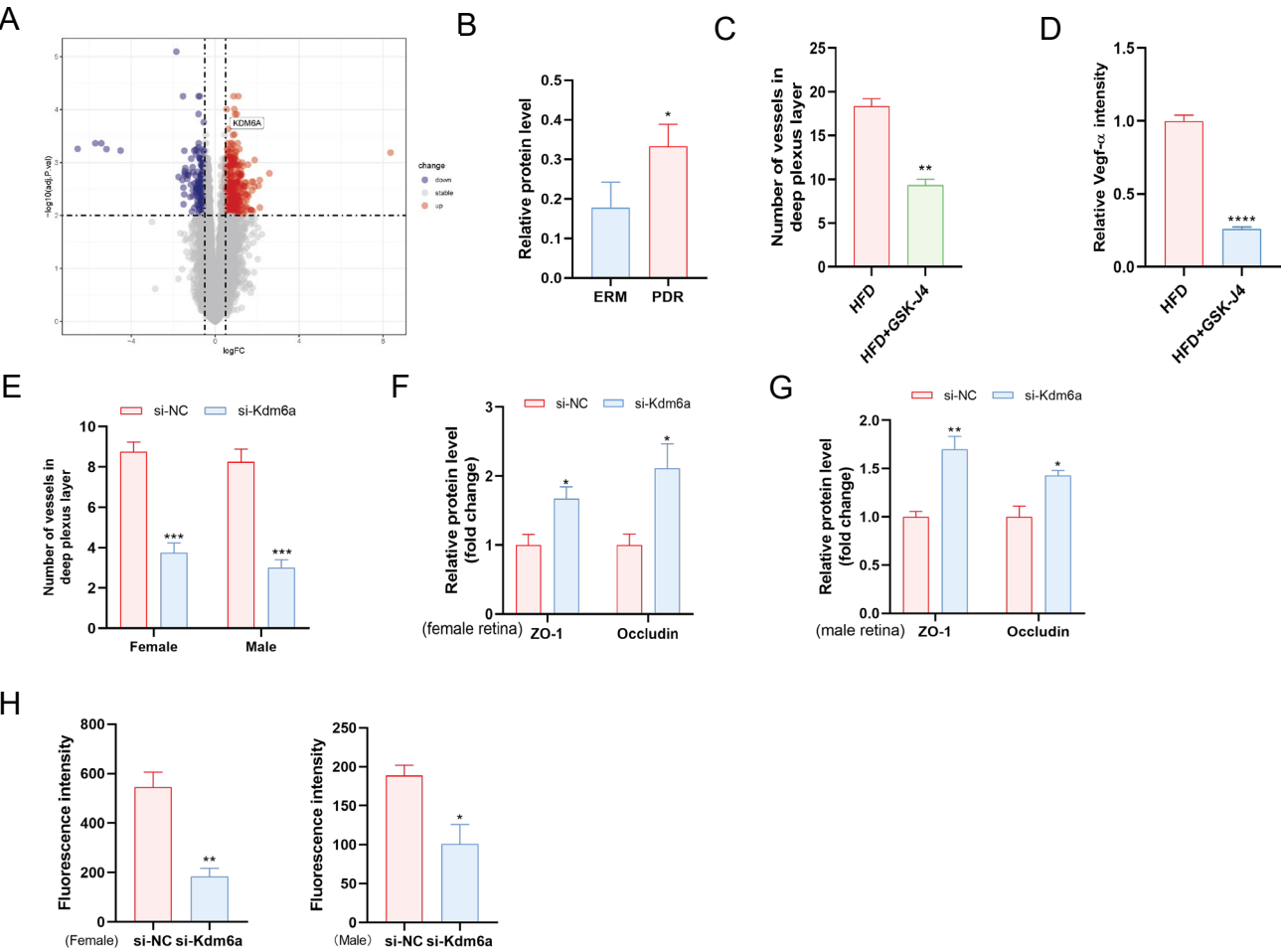

Supplement: Supplementary file 1 — Supplementary Figure 1 [file 41419_2026_8816_MOESM1_ESM.pdf]

# Supplementary Figure 2

A

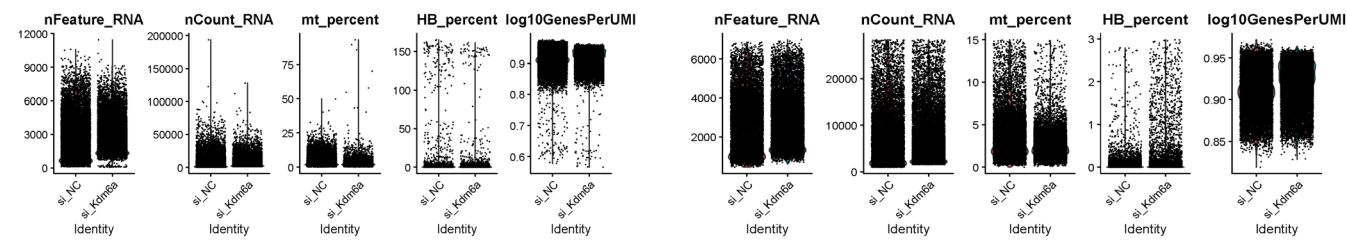

B

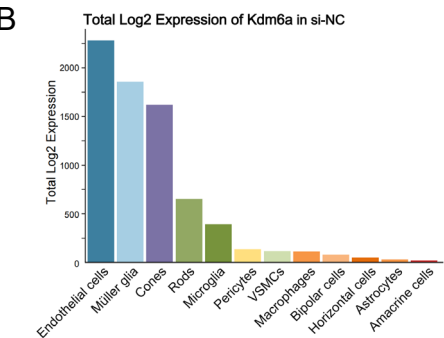

C

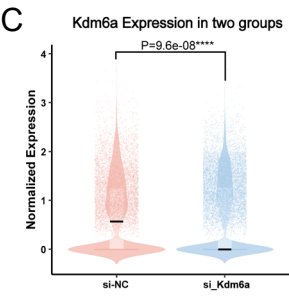

D

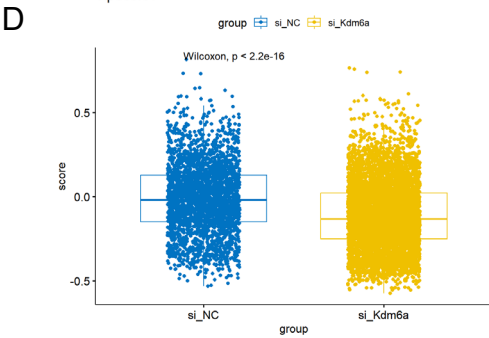

E

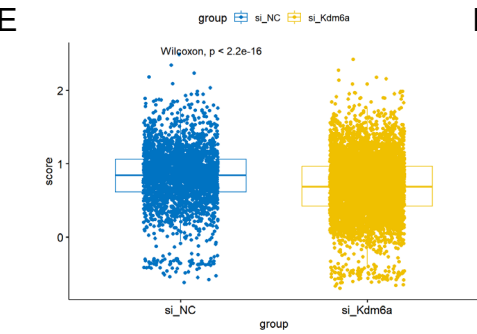

F

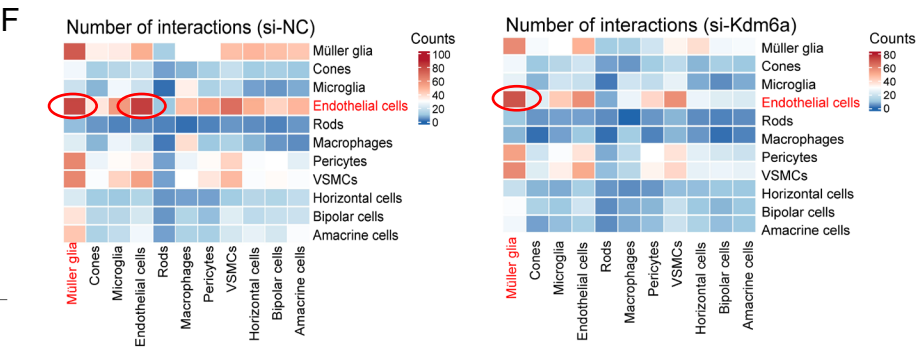

G

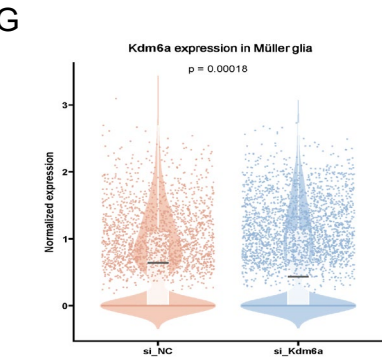

H

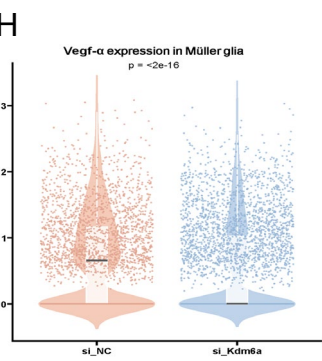

I

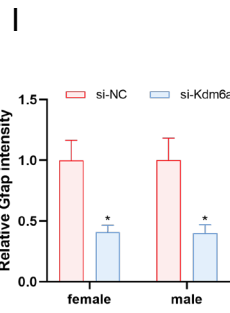

J

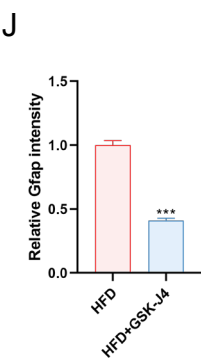

Supplement: Supplementary file 2 — Supplementary Figure 2 [file 41419_2026_8816_MOESM2_ESM.pdf]

# Supplementary Figure 3

A

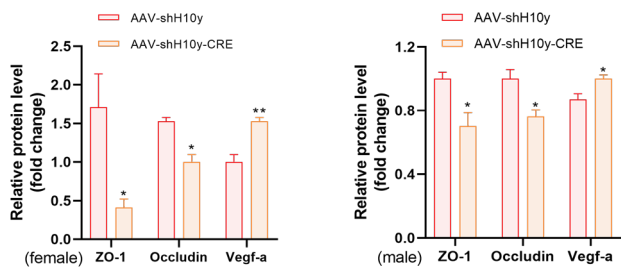

B

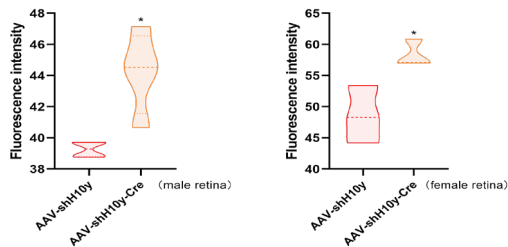

C

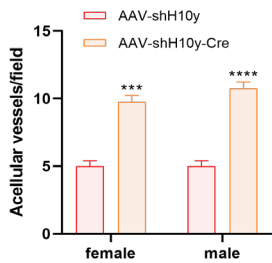

Supplement: Supplementary file 3 — Supplementary Figure 3 [file 41419_2026_8816_MOESM3_ESM.pdf]

# Supplementary Figure 4

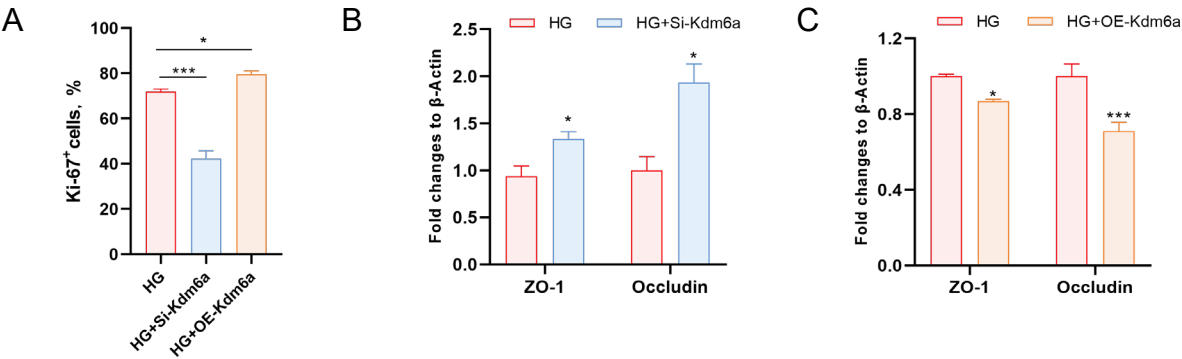

Supplement: Supplementary file 4 — Supplementary Figure 4 [file 41419_2026_8816_MOESM4_ESM.pdf]

Supplementary Figure 5

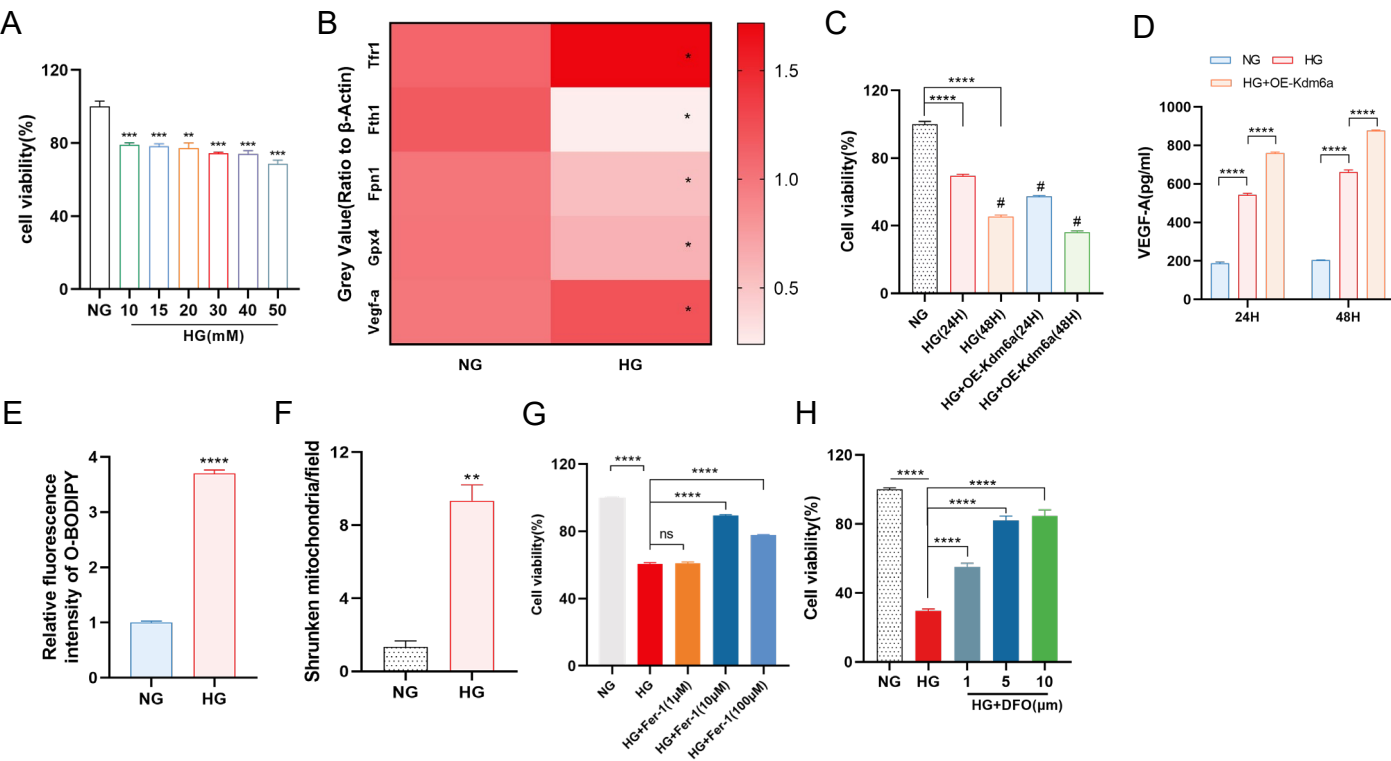

Supplement: Supplementary file 5 — Supplementary Figure 5 [file 41419_2026_8816_MOESM5_ESM.pdf]

# Supplementary Figure 6

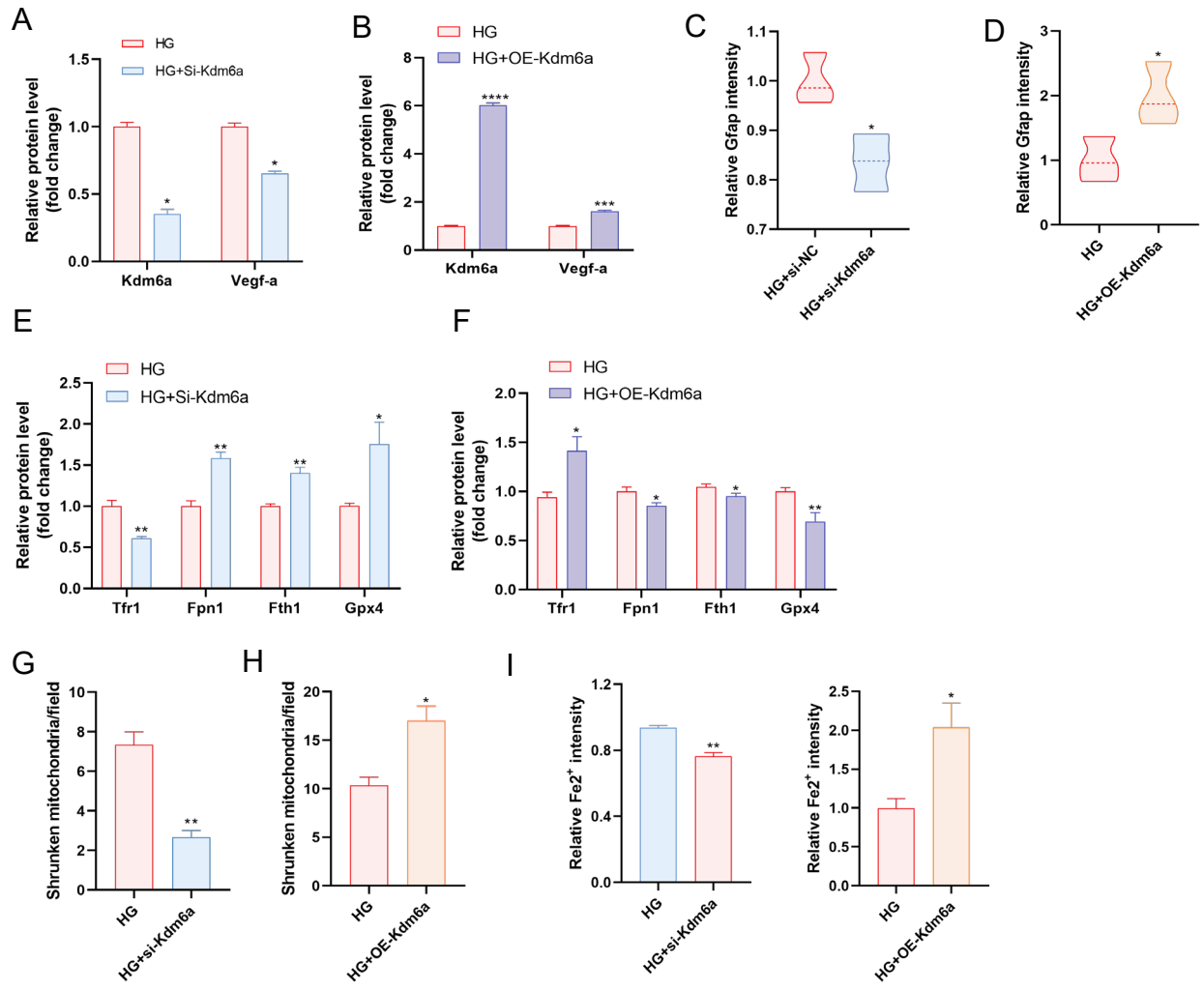

Supplement: Supplementary file 6 — Supplementary Figure 6 [file 41419_2026_8816_MOESM6_ESM.pdf]

# Supplementary Figure 7

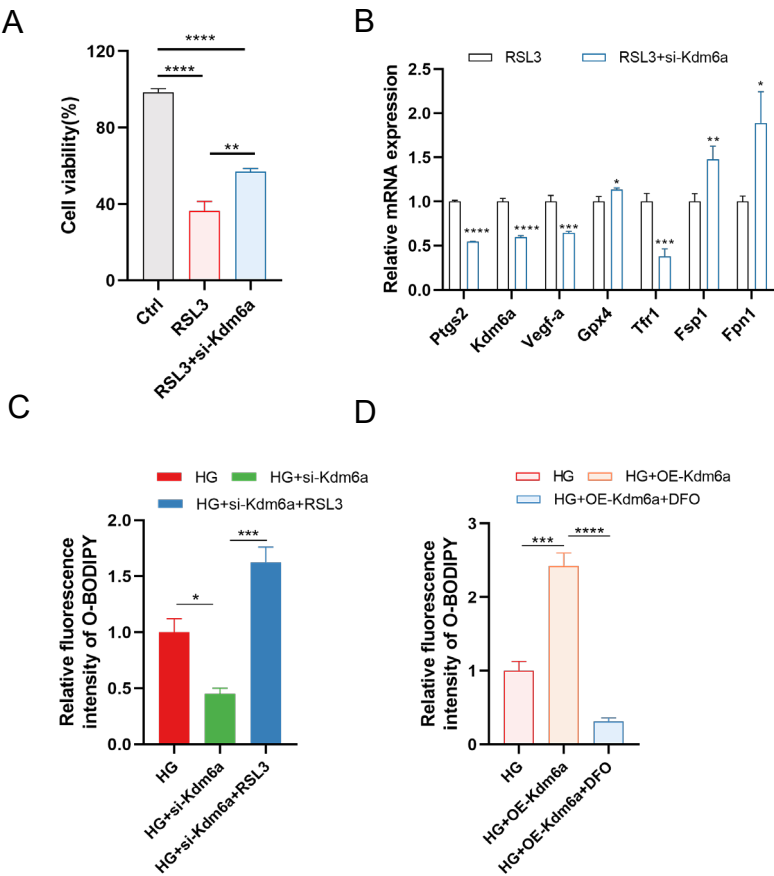

Supplement: Supplementary file 7 — Supplementary Figure 7 [file 41419_2026_8816_MOESM7_ESM.pdf]
