# Supplementary material for: KDM6A promotes diabetic retinopathy via H3K27me3-dependent ferroptosis in Müller cells
Source: Cell Death Dis. 2026 Apr 29;17(1):571. doi: 10.1038/s41419-026-08816-9 (PMC13265779; doi:10.1038/s41419-026-08816-9)
Supplement: Supplementary file 8 — Supplementary Material [file 41419_2026_8816_MOESM8_ESM.docx]

**Supplementary Figure Legends**

**Supplementary Figure 1**

A. Volcano plot of differentially expressed genes (DEGs) between NPDR and PDR groups.

B. Quantification of Kdm6a protein levels in ERM and PDR groups. n=3.

C. Quantitative analysis of deep vascular plexus density (CD31+ immunofluorescence) in retinal sections HFD mice following GSK-J4 or DMSO treated, n=3.

D. Quantitative analysis of VEGF-A intensity in retinal sections HFD mice following GSK-J4 or vehicle treated, n=3.

E. Quantitative analysis of deep vascular plexus density (CD31+ immunofluorescence) in retinal sections from male and female db/db mice following intravitreal injection of si-NC or si- Kdm6a, n=3.

F. Quantification of retinal tight junction proteins ZO-1 and Occludin in female db/db mice following si-NC or si-Kdm6a injection. n = 3.

G. Quantification of retinal tight junction proteins ZO-1 and Occludin in male db/db mice following si-NC or si-Kdm6a injection. n = 3.

H. Quantification of Evans Blue vascular leakage fluorescence intensity in male and female *db/db* mice following intravitreal injection of si-NC or si- Kdm6a. n=3. Data = mean ± SEM; **P*<0.05, ***P*<0.01, ****P*<0.001, unpaired two-tailed Student's t-test.

**Supplementary Figure 2**

A. Sc-RNA-seq QC metrics: Pre- vs post-filtering in retinal cells of si-NC/si- Kdm6a -injected db/db mice.

B. Bar plots of Kdm6a expression distribution in major retinal cell clusters.

C. Violin plots showing KDM6A expression across retinal cell types in the si-NC and si-KDM6A groups.

D. Tip cell signature scoring in endothelial subclusters from single-cell data (si-NC vs. si- Kdm6a)

E. Stalk cell signature scoring in endothelial subclusters from single-cell data (si-NC vs. si- Kdm6a)

F. Cell-cell communication networks in the diabetic retina of si-NC- and si-Kdm6a-injected db/db mice.

G. Violin plots of Kdm6a mRNA expression in Müller cells from si-NC and si-Kdm6a groups based on scRNA-seq.

H. Violin plots of Vegf-α mRNA expression in Müller cells from si-NC and si-Kdm6a groups based on scRNA-seq.

I. Quantification of GFAP fluorescence intensity in retinal sections from male and female db/db mice following intravitreal injection of si-NC or si-Kdm6a (n=3).

J. Quantification of GFAP fluorescence intensity in HFD mice following GSK-J4 or vehicle treated. Scar bar= 50μm, n=3.Data = mean ± SEM. Data = mean ± SEM, **p*<0.05, *** *p* <0.001, unpaired two-tailed Student's t-test.

**Supplementary Figure 3**

A. Quantification of retinal tight junction proteins ZO-1 and Occludin in diabetic Kdm6aki/ki and Kdm6aki/y mice injected with AAV-shH10y or AAV-shH10y-Cre( n = 3).

B. Quantification of retinal Evans Blue fluorescence intensity in diabetic Kdm6aki/ki and Kdm6aki/y mice following intravitreal injection of AAV-shH10y or AAV-shH10y-Cre (n = 3).

C. Quantification of acellular capillaries in PAS-stained retinas of male and female mice injected with AAV-shH10y or AAV-shH10y-Cre (n = 3). Data = mean ± SEM, **P*<0.05, ***P*<0.01, unpaired two-tailed Student's t-test.

**Supplementary Figure 4**

A. Ki67^+^ cell quantification in HG-exposed endothelia cell co-cultured with Müller cell under: (1) HG (30mM) (2) HG +si- Kdm6a (3) HG + OE- Kdm6a, n=3.

B. ZO-1 and Occludin protein levels in endothelia co-cultured with HG or HG+si-Kdm6a treated Müller cells, n=3.

C. ZO-1 and Occludin protein levels in endothelia co-cultured with HG or HG+OE- Kdm6a treated Müller cells, n=3. Data = mean ± SEM, **P*<0.05, ****P*<0.001, *****P*<0.0001, unpaired two-tailed Student's t-test.

**Supplementary Figure 5**

A. Viability of Müller cells after 48h exposure to different glucose concentration, n=6.

B. The protein level of Tfr1, Fth1, Fpn1, Gpx4 and VEGF-A in Müller cell after HG stimulation, n=4.

C. CCK-8 assay showing the effect of Kdm6a overexpression on cell viability in Müller cells under HG conditions (n=6).

D. ELISA analysis of VEGF-A concentration in the supernatant of Müller cells treated with NG, HG for 24 h or 48 h, or HG combined with Kdm6a overexpression for 24 h or 48 h, n=3.

E. Quantification of O-BODIPY-positive intensity in Müller cell under NG or HG condition, n=3.

F. Quantification of shrunken mitochondria in Müller cell under NG or HG condition, n=3.

G. Cell viability of Müller cells under high glucose (HG) conditions with or without Fer-1, n = 6.

H. Cell viability of Müller cells under high glucose (HG) conditions with or without DFO, n=6. Data = mean ± SEM, **P*<0.05, ***P*<0.01****P*<0.001, *****P*<0.0001, one-way ANOVA followed by Tukey’s post hoc test and unpaired two-tailed Student's t-test.

**Supplementary Figure 6**

A. The protein level of Kdm6a and Vegf-α in Müller cell treated with HG or HG+si- Kdm6a, n=3.

B. Protein expression of Kdm6a and VEGF-A in Müller cells under HG or HG+si-Kdm6a treatment. n = 3.

C. Quantification of GFAP immunofluorescence intensity in Müller cells treated with HG or HG+si-Kdm6a. n = 6.

D. Quantification of GFAP immunofluorescence intensity in Müller cells treated with HG or HG+OE-Kdm6a. n = 6.

E. Protein expression of Tfr1, Fth1, Fpn1 and Gpx4 in Müller cell treated with HG or HG+ si- Kdm6a, n=3.

F. The protein level of Tfr1, Fth1, Fpn1 and Gpx4 in Müller cell treated with HG or HG+OE- Kdm6a, n=3.

G. Quantification of shrunken mitochondria in Müller cells treated with HG or HG+ si- Kdm6a, n=3.

H. Quantification of shrunken mitochondria in Müller cells treated with HG or HG+ OE- Kdm6a, n=3.

I. Quantification of Fe2^+^ in Müller cells treated with HG, HG+si-Kdm6a or HG+ OE- Kdm6a, n=6. Data = mean ± SEM, **P*<0.05, ***P*<0.01****P*<0.001, *****P*<0.0001, one-way ANOVA followed by Tukey’s post hoc test and unpaired two-tailed Student's t-test.

**Supplementary Figure 7**

A. Cell viability of Müller cells treated with RSL3 in the presence or absence of si-Kdm6a, n=6.

B. The mRNA level of Ptgs2, Kdm6a, Vegf-α, Tfr1, Fth1, Fpn1 and Gpx4 in Müller cell treated RSL3 in the presence or absence of si-Kdm6a, n=3.

C. Quantification of O-BODIPY fluorescence intensity in Müller cells treated with HG, HG+si-Kdm6a, or HG+si-Kdm6a +RSL3, n=3.

D. Quantification of O-BODIPY fluorescence intensity in Müller cells treated with HG, HG+OE-Kdm6a, or HG+OE-Kdm6a +DFO, n=3. **P*<0.05, ***P*<0.01****P*<0.001, *****P*<0.0001, one-way ANOVA followed by Tukey’s post hoc test and unpaired two-tailed Student's t-test.

**Supplementary Table 1. Primer sequences**

| **gene name** | **Forward** | **Reverse** |
| --- | --- | --- |
| Kdm6a | CGGGCGGACAAAAGAAGAAC | CATAGACTTGCATCAGATCCTCC |
| Ptgs2 | TGCACTATGGTTACAAAAGCTGG | TCAGGAAGCTCCTTATTTCCCTT |
| Gpx4 | ATAAGAACGGCTGCGTGGTGAAG | TAGAGATAGCACGGCAGGTCCTTC |
| Tfr1 | GCTGTCCCTGACAAAACGGT | CGGAAGGACGGTCTTCATGTG |
| Fpn1 | ACCAAGGCAAGAGATCAAACC | AGACACTGCAAAGTGCCACAT |
| Fth1 | CAAGTGCGCCAGAACTACCA | GCCACATCATCTCGGTCAAAA |
| Vegf-α | GCACATAGAGAGAATGAGCTTCC | CTCCGCTCTGAACAAGGCT |
| Acsl4 | CCTTTGGCTCATGTGCTGGAAC | GCCATAAGTGTGGGTTTCAGTAC |
| Lpcat3 | CCATCTCTTCCACACCTTCACG | GGATGAGGAACTGAAGCACGAC |
| Fads2 | TTCCTGGAGAGCCACTGGTTTG | GAAGAAGGACTGCTCCACATTGC |
| β-Actin | TTCGTTGCCGGTCCACACCC | GCTTTGCACATGCCGGAGCC |

**Supplementary Table 2. Antibody list**

| **Antibody** | **Catalog** |
| --- | --- |
| Kdm6a | PA5-31828, Invitrogen |
| GFAP | ab68428, Abcam |
| CD31 | ab76533, Abcam |
| Gpx4 | T56959, Abmart |
| Tfr1 | T56618, Abmart |
| Fpn1 | TD13561, Abmart |
| Fth1 | T55648, Abmart |
| Vegf-α | 19003-1-AP, Proteintech |
| ZO-1 | 21773-1-AP, Proteintech |
| Occludin | ab224526, Abcam |
| β-Actin | AC026, ABclonal |
| HRP-conjugated Rabbit | AS092, Abclonal |
| Alexa Fluor® 488 | ab150077, Abcam |
| Alexa Fluor® 594 | ab150080, Abcam |
