# Supplementary material for: KDM6A promotes diabetic retinopathy via H3K27me3-dependent ferroptosis in Müller cells
Source: Cell Death Dis. 2026 Apr 29;17(1):571. doi: 10.1038/s41419-026-08816-9 (PMC13265779; doi:10.1038/s41419-026-08816-9)

Fig1C

Kdm6a

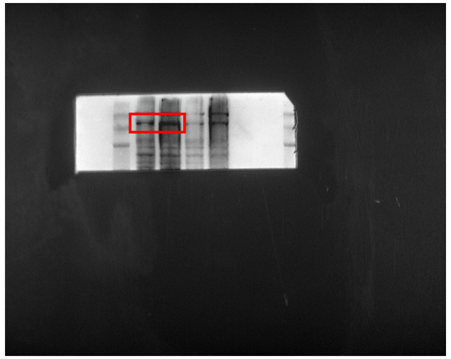

Actin

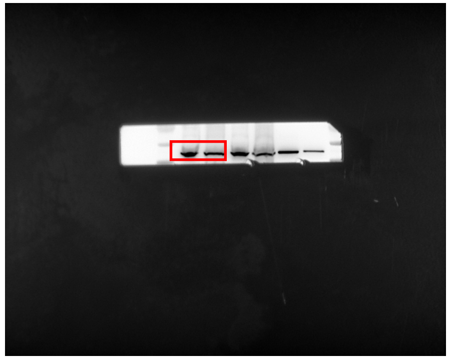

Fig1I-female

ZO1

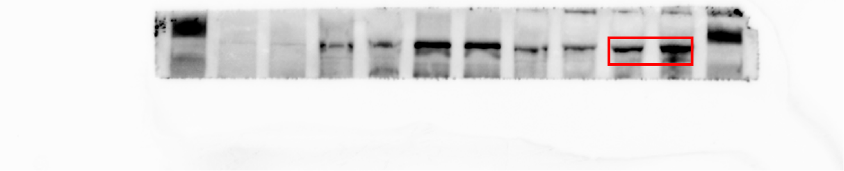

Occludin

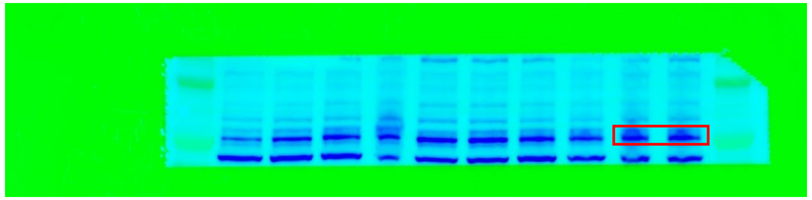

Actin

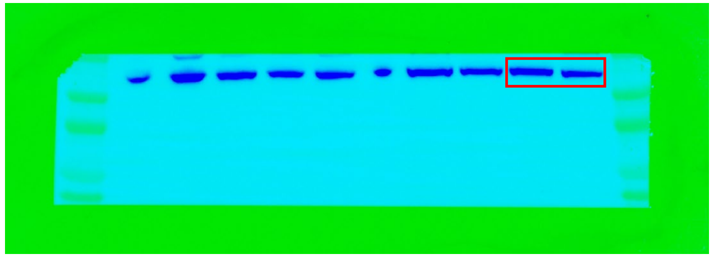

Fig1I-Male

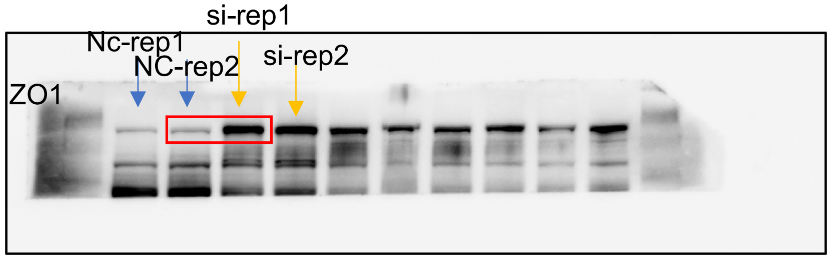


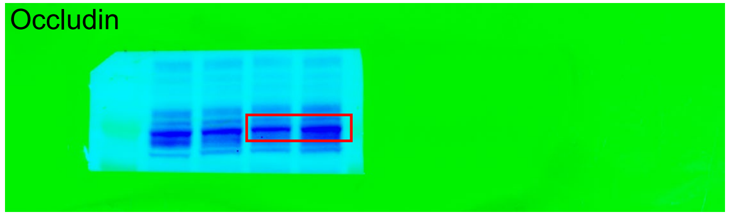


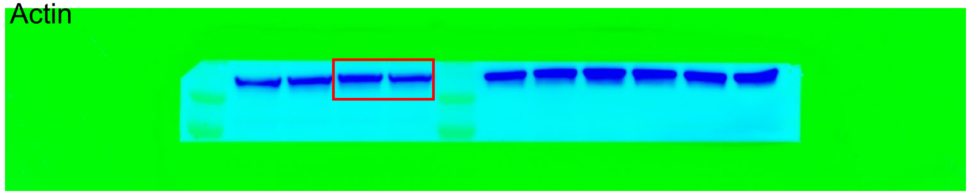

Fig3I

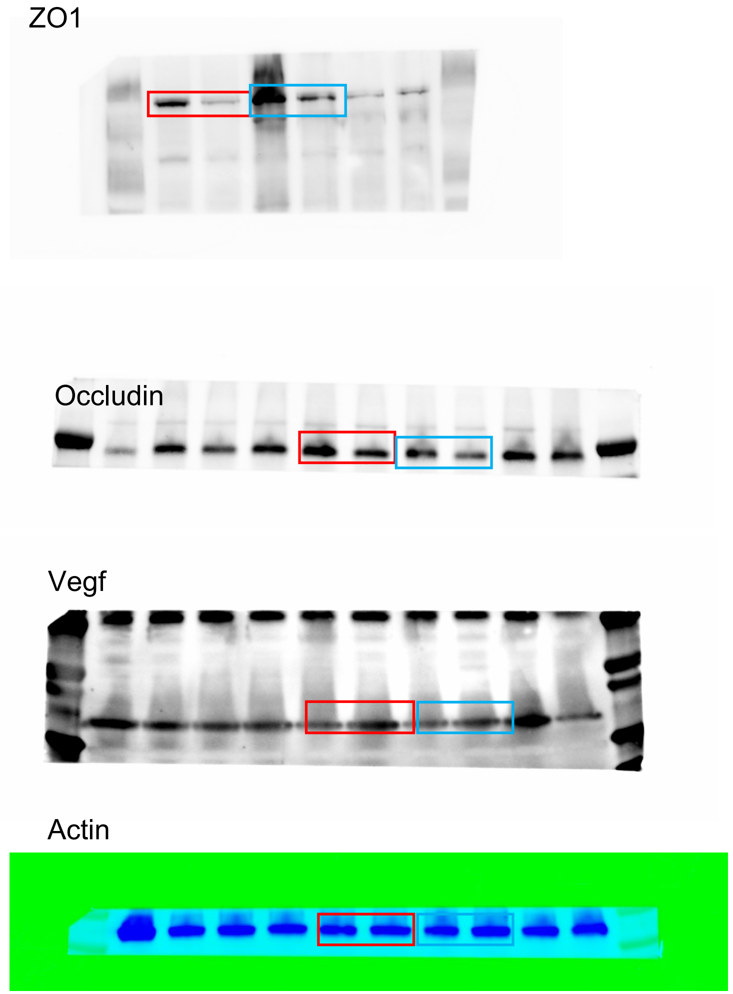
ZO1
Occludin
Vegf
Actin

Fig4I

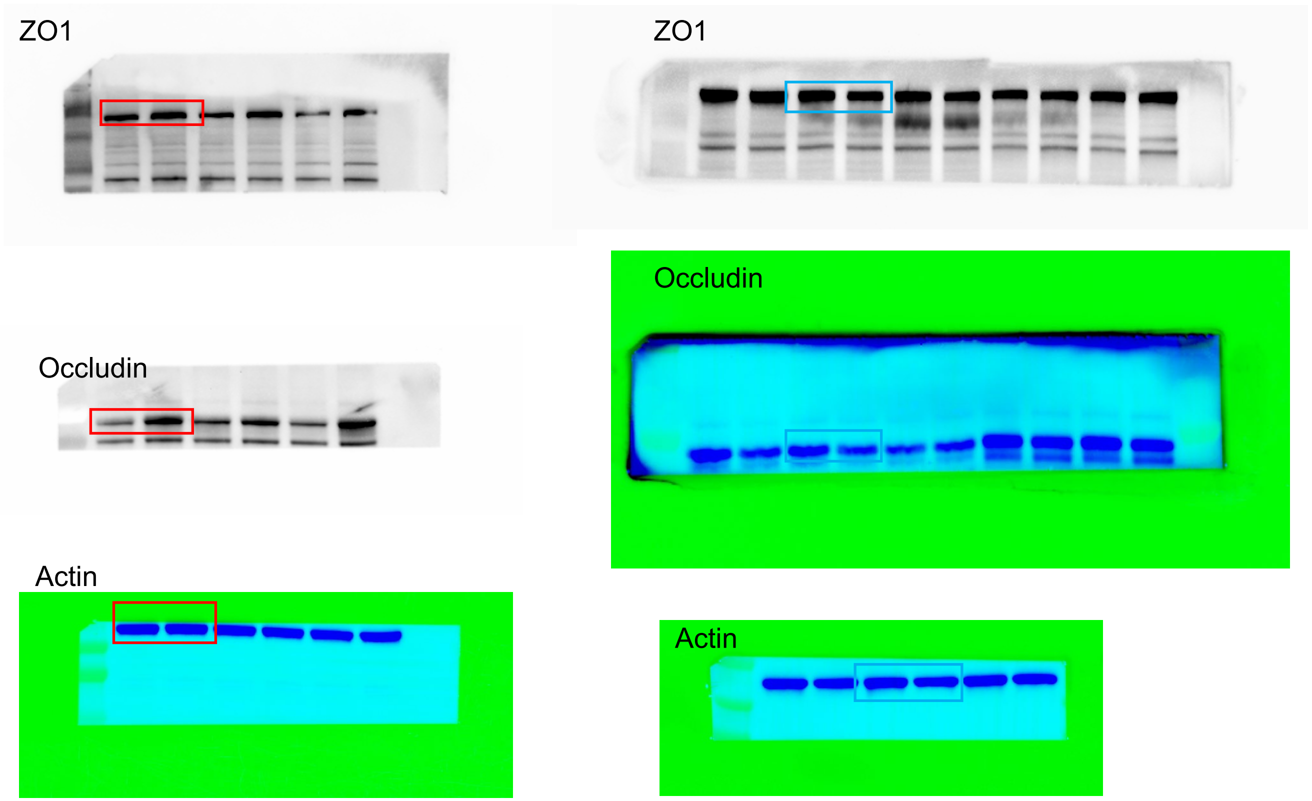
ZO1
ZO1
Occludin
Occludin
Actin
Actin

Fig5C

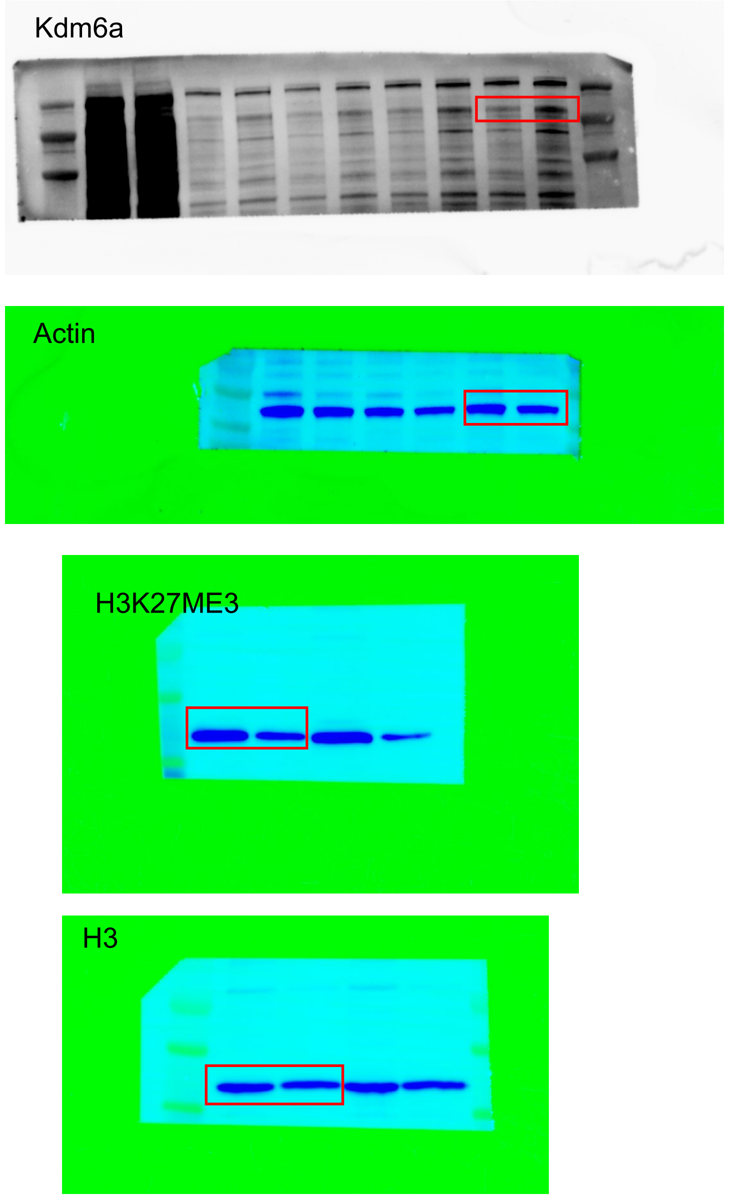


Fig5J

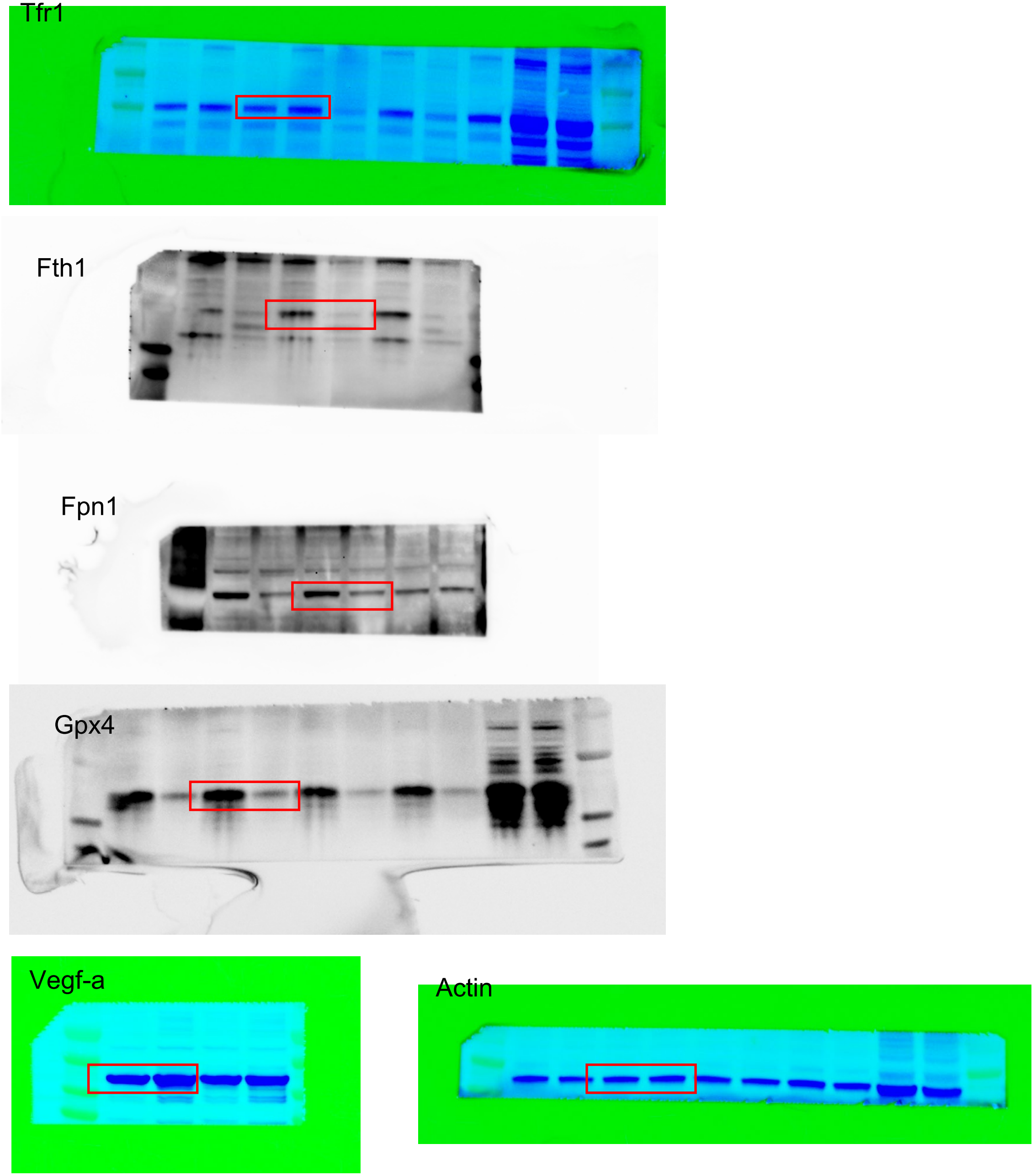

Fig6B

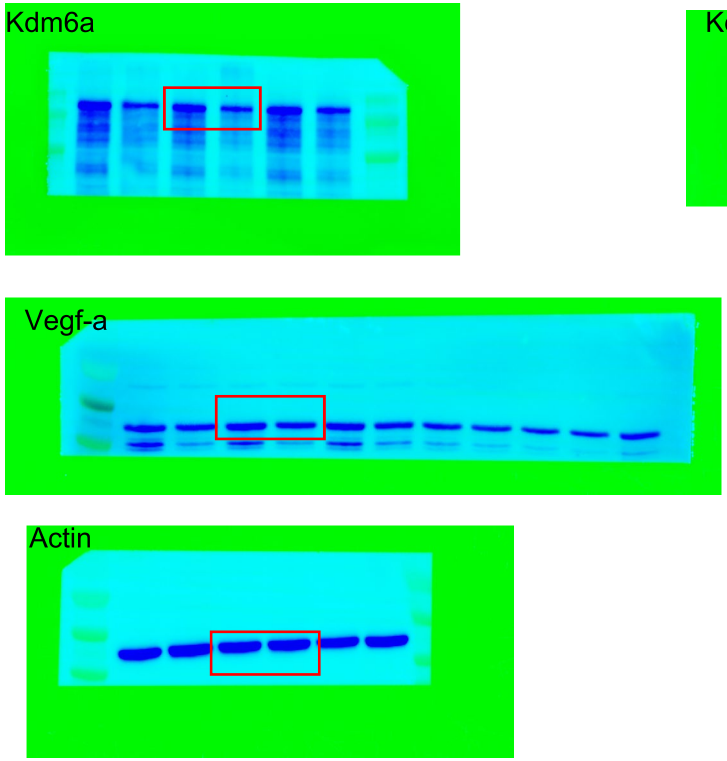


Fig6C

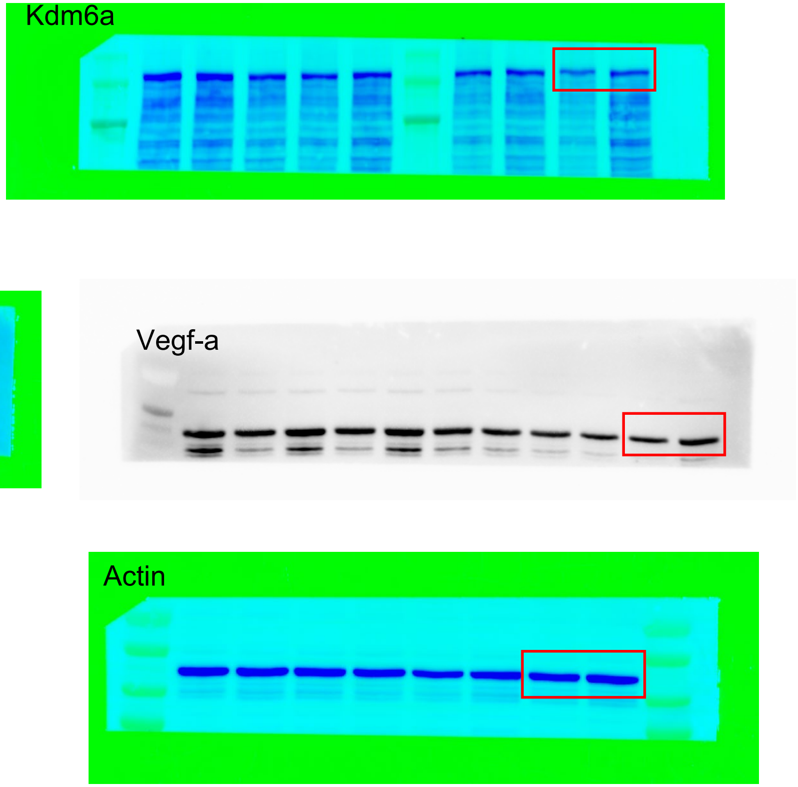

Fig6H-HG VS HG+si-Kdm6a

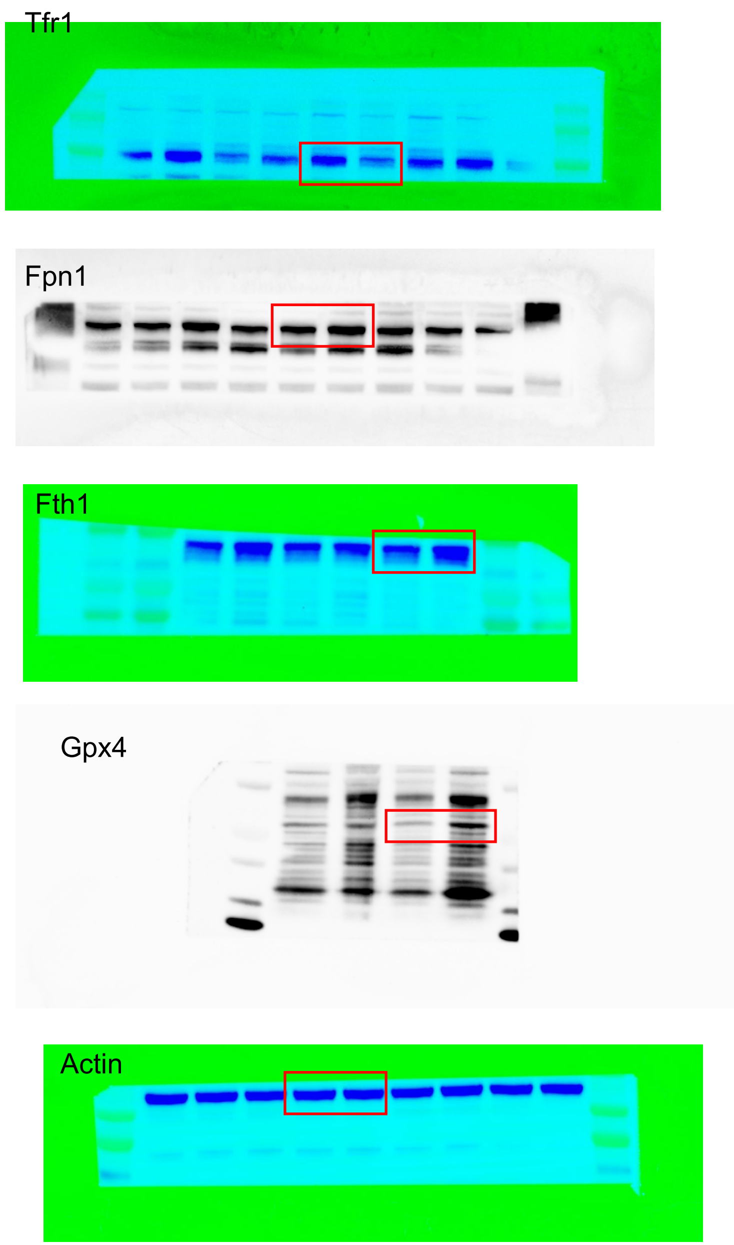


Fig6H-HG VS HG+OE-Kdm6a

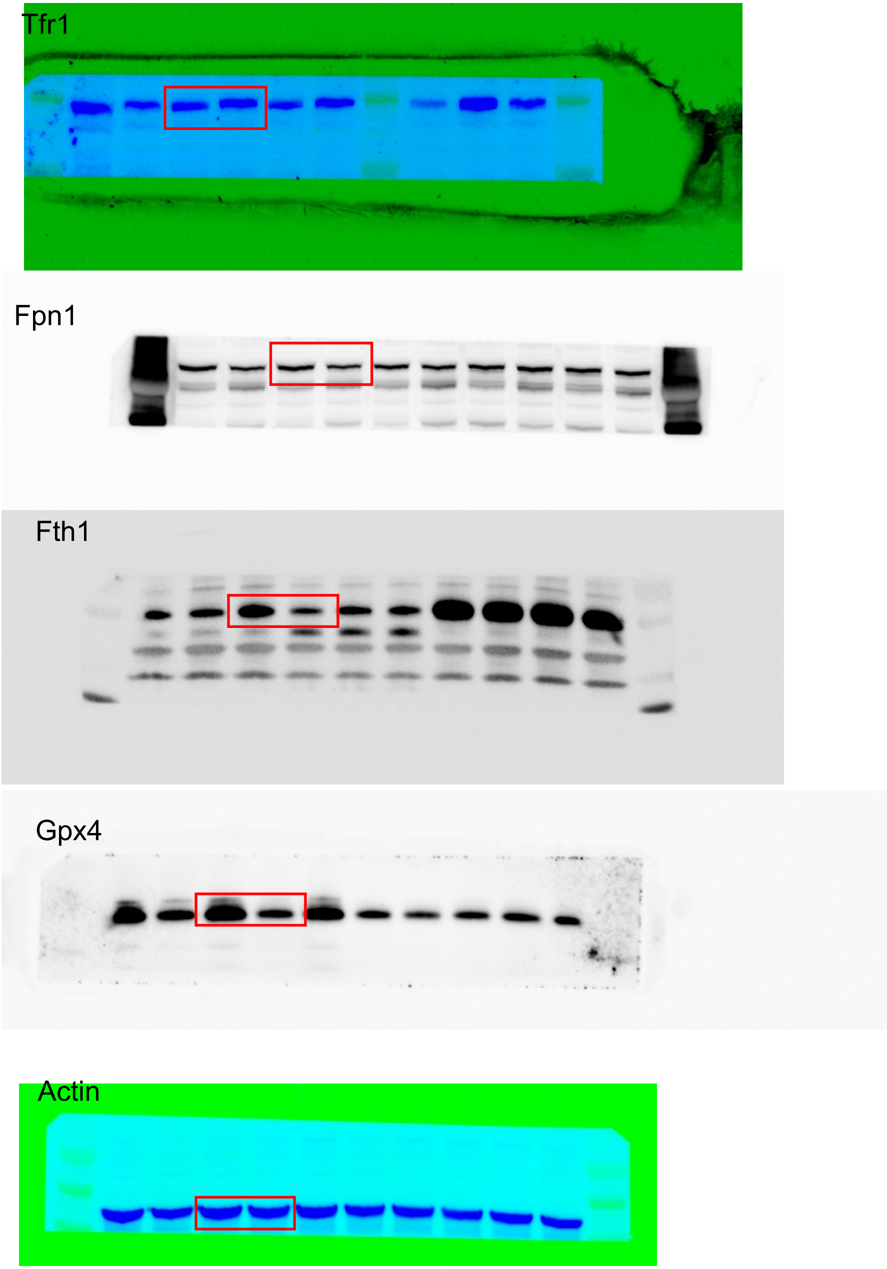

Supplement: Supplementary file 9 — Full Scans of Western Blots [file 41419_2026_8816_MOESM9_ESM.pdf]
